# Recent Advances Regarding the Molecular Mechanisms of Triterpenic Acids: A Review (Part I)

**DOI:** 10.3390/ijms23147740

**Published:** 2022-07-13

**Authors:** Marius Mioc, Andreea Milan, Daniel Malița, Alexandra Mioc, Alexandra Prodea, Roxana Racoviceanu, Roxana Ghiulai, Andreea Cristea, Florina Căruntu, Codruța Șoica

**Affiliations:** 1 Department of Pharmaceutical Chemistry, Faculty of Pharmacy, “Victor Babes” University of Medicine and Pharmacy Timisoara, Eftimie Murgu Sq., No. 2, 300041 Timisoara, Romania; marius.mioc@umft.ro (M.M.); andreea.milan@umft.ro (A.M.); alexandra.ulici@umft.ro (A.P.); babuta.roxana@umft.ro (R.R.); roxana.ghiulai@umft.ro (R.G.); andreea.cristea.umfvbt@gmail.com (A.C.); codrutasoica@umft.ro (C.Ș.); 2 Research Centre for Pharmaco-Toxicological Evaluation, “Victor Babes” University of Medicine and Pharmacy Timisoara, Eftimie Murgu Sq., No. 2, 300041 Timisoara, Romania; 3 Department of Radiology, “Victor Babes” University of Medicine and Pharmacy Timisoara, 300041 Timisoara, Romania; 4 Department of Anatomy, Physiology, Pathophysiology, Faculty of Pharmacy, “Victor Babes” University of Medicine and Pharmacy Timisoara, Eftimie Murgu Sq., No. 2, 300041 Timisoara, Romania; 5 Department of Medical Semiology II, Faculty of Medicine, “Victor Babeş” University of Medicine and Pharmacy Timisoara, 2 Eftimie Murgu Street, 300041 Timisoara, Romania; caruntu.florina@umft.ro

**Keywords:** triterpenic acid, asiatic acid, oleanolic acid, ursolic acid, molecular target, molecular mechanism

## Abstract

Triterpenic acids are phytocompounds with a widespread range of biological activities that have been the subject of numerous in vitro and in vivo studies. However, their underlying mechanisms of action in various pathologies are not completely elucidated. The current review aims to summarize the most recent literature, published in the last five years, regarding the mechanism of action of three triterpenic acids (asiatic acid, oleanolic acid, and ursolic acid), corelated with different biological activities such as anticancer, anti-inflammatory, antidiabetic, cardioprotective, neuroprotective, hepatoprotective, and antimicrobial. All three discussed compounds share several mechanisms of action, such as the targeted modulation of the PI3K/AKT, Nrf2, NF-kB, EMT, and JAK/STAT3 signaling pathways, while other mechanisms that proved to only be specific for a part of the triterpenic acids discussed, such as the modulation of Notch, Hippo, and MALAT1/miR-206/PTGS1 signaling pathway, were highlighted as well. This paper stands as the first part in our literature study on the topic, which will be followed by a second part focusing on other triterpenic acids of therapeutic value.

## 1. Introduction

**Figure 1:**
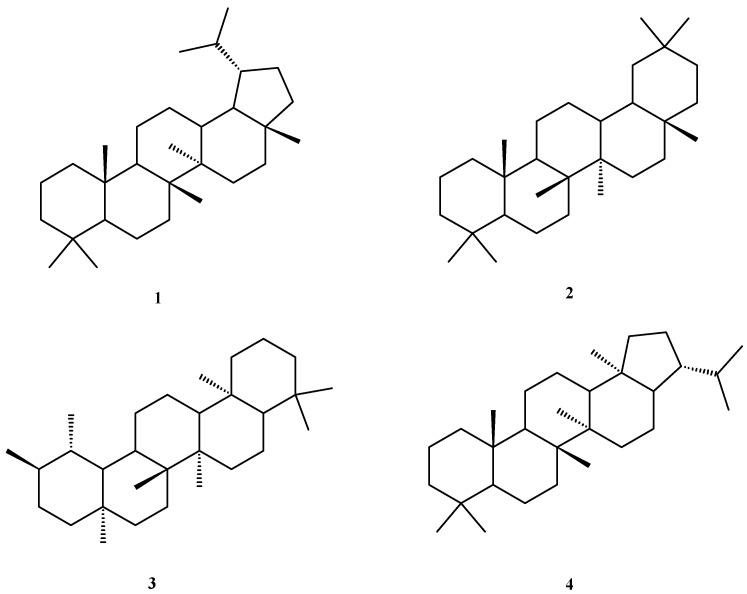
The chemical structures of lupane (**1**), oleanane (**2**), ursane (**3**), and hopane (**4**) scaffolds.

Ever since the discovery by Pisha et al. in 1995 of the selective anti-melanoma efficiency of betulinic acid, pentacyclic triterpenes have entered the scrutiny of numerous research groups in order to identify their ability to fight various diseases in particular malignant tumors. Triterpenes represent the largest group of terpenoids, natural compounds isolated from various plants, animals, microorganisms, and oils [1], which contain various numbers of isoprene units; in turn, triterpenes can be subdivided according to the number of condensed rings in their structure in acyclic, mono-, di-, tri-, tetra-, and pentacyclic subgroups. Several representatives have shown remarkable antiproliferative effects against melanoma, neuroblastoma, leukemia, and brain tumors, alongside other types of cancer [2,3]. Pentacyclic triterpenes may contain lupane, oleanane, ursane, or hopane scaffolds (Figure 1) widely found in various plant organs and numerous vegetal species; they stand as the most thoroughly investigated triterpene group due to their diverse pharmacological effects. Triterpenic acids with lupane, oleane, and ursane structures have been extracted from numerous plant families and tested for biological effects; they were revealed as strong anticancer, anti-inflammatory, and antioxidant agents. In addition, the literature also reported their immunomodulatory and antimicrobial effects and, more recently, their ability to fight type 2 diabetes has been emphasized. Furthermore, considering their wide range of pharmacological activities, research was conducted in order to identify the key genes involved in their biosynthetic pathways so that improved biotechnological approaches could be designed and applied for their production [4]. For example, several investigations point out that the triterpenic scaffold can inhibit topoisomerases I and IIα by competing with DNA for the topoisomerase binding site [1,5,6].

One major therapeutic benefit when using pentacyclic triterpenes as pharmacological agents is their selective biological effects, which largely prevent the occurrence of side effects; pharmacological selectivity is particularly beneficial when the triterpenes are used as cytotoxic agents against tumor cells keeping healthy cells unharmed. This behavior comes in sharp opposition to synthetic drugs, which act undifferentiated on normal and tumor cells, therefore triggering severe side effects with potential lethal consequences. The combination of therapeutic efficiency with the lack of toxic activity makes pentacyclic triterpenes attractive alternatives to conventional treatments; however, in order to be accepted as therapeutic agents, triterpenes must undergo several testing stages in order to reveal their efficacy/toxicity ratio, which reflects their safety for human use.

One well-known drawback of these phytocompounds is the reduced bioavailability that limits their use. To overcome this limitation, pentacyclic triterpenoids have been subjected to numerous derivatizations, which led to some derivatives with superior activity compared to parent compounds [7]. For instance, Spivak et al. [8] synthesized a series of cationic conjugates of betulin and several triterpenic acids, which specifically targeted the mitochondria of several cancer cells in vitro, showing a significant increase in cytotoxicity (more than 100 fold for the betulinic acid derivative compared to betulinic acid) compared to natural compounds.

The investigation of the mechanism of action (MOA) before entering clinical trials is considered by some researchers as an essential step in the development process of a new drug [9] since it may provide significant information on the drug’s safety, adequate posology, and patient therapeutic response [10]. However, since the mechanistic aspects are not mandatory for drug approval, some drug developers may be tempted to bypass this investigation due to economic reasons [11], although it may perhaps lead to the failure of the drug development process, even in its advanced stages (Phase III clinical trial). Despite the fact that many drugs are currently used without proper knowledge of their MOA [12], clarification of this issue may prevent the late-stage failure in drug development, orientate the design of clinical trials, and avoid potential side effects [13]; moreover, based on the knowledge of MOA, researchers are able to design drug analogs with higher efficacy and lower adverse effects [14]. Therefore, in the long run, the identification of MOA very early in the drug’s development process may actually increase the success of drug approval and, more importantly, produce therapeutic benefits in patient outcome.

**Figure 2:**
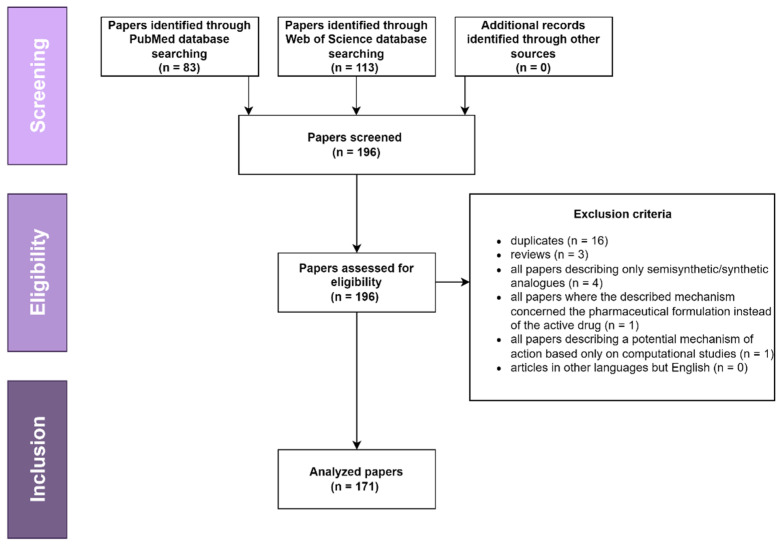
Flow diagram describing the data selection process.

It has become very clear that, considering their very large plethora of biologic effects, triterpenic acids presumably act on multiple pathways; indeed, there are many reviews published in the past 10 years regarding their mechanism of action responsible for various activities, such as anticancer [15,16,17,18,19,20,21,22], immunomodulatory [23,24], antidiabetic [25,26,27,28,29,30], anti-inflammatory [31], cardioprotective [32], antiurolithic [33], antiviral [34], and wound healing [35,36]. This review aims to summarize the most recent (last five years) discoveries on triterpenic acids’ mechanisms of action in order to provide scientists with exhaustive knowledge, which in turn might propel and diversify future studies. As the authors intended to offer a comprehensive review on the topic and the literature published in recent years is vast, the material from the following literature survey had to be divided into two parts. The current paper represents the first part and depicts the most recent developments regarding the underlying MOA of asiatic acid, oleanolic acid, and ursolic acid. Two major databases were screened, PubMed and Web of Science, using as key search terms the main triterpenic acids’ names combined with “mechanism”; following the cross-examination of results by two experienced researchers, the following papers were included: (1) in vitro studies; (2) in vivo studies; (3) studies describing the mechanism of action of natural triterpenic acids; and the following types of papers were eliminated: (1) duplicates; (2) reviews; (3) all papers describing only semisynthetic/synthetic analogues; (4) all papers where the described mechanism concerned the pharmaceutical formulation instead of the active drug; (5) all papers describing a potential mechanism of action based only on computational evaluation; (6) articles in other languages but English (Figure 2). 

## 2. Asiatic Acid

**Figure 3:**
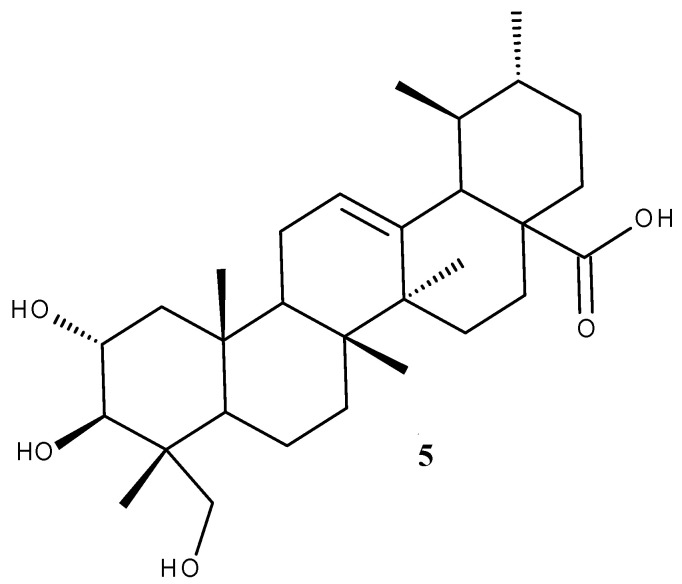
The chemical structure of asiatic acid (**5**).

Asiatic acid (AA, Figure 3) is a pentacyclic triterpenoid extracted from the tropical species *Centella asiatica* used in Traditional Chinese and Indian Ayurvedic medicine as treatment for various skin conditions including wound healing, gastrointestinal and genitourinary diseases, anxiety, and cognitive disorders [37]; in addition, it can be found in numerous other plant species. AA is the aglycone of asiaticoside which, next to madecassoside, represents the biomarker components of *C. asiatica* extract due to their high content compared to other components; as such, they can be used as indicators for the quality analysis of the plant extract [38].

**Figure 4:**
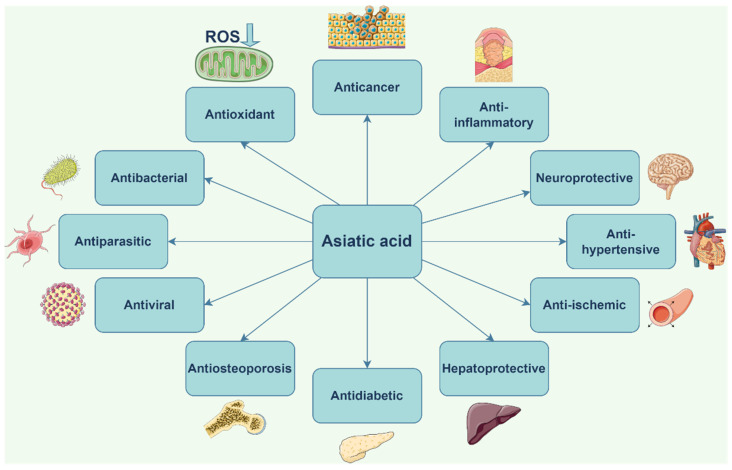
Biological activities of asiatic acid.

Two excellent reviews on AA were published in 2018 by Meeran et al. and Lv et al. [39,40] comprehensively describing its biosynthesis, pharmacokinetics, and pharmacodynamics properties as well as its molecular mechanisms. AA was revealed as a pluripotent biologic agent with a large plethora of therapeutic properties that have become useful in various conditions (Figure 4).

### 2.1. Anticancer Activity

Several studies have been conducted on the potential use of AA against breast cancer, resulting in the identification of certain molecular targets. AA was tested on the estrogen receptor (+) MCF7 as well as on the estrogen receptor (-) MDA-MB-231 breast cancer cell lines, inhibiting both cell lines but acting stronger against MDA-MB-231 cells [41]. In terms of molecular mechanism, it was found that AA interferes with WAVE3 expression; WAVE3 (Wiskott-Aldrich syndrome verprolin-homologous 3) is a member of the WASP/WAVE family of proteins, which play essential roles in cell shape and motility. In particular, WAVE3 is critically involved in the invasion of cancer cells due to its up-regulation in various types of cancer, which is associated with aggressive progression and metastasis [42]. The results showed that the compound’s ability to induce apoptosis in MDA-MB-231 cells was reduced after cell transfection with pcDNA3.1-WAVE3 plasmid, which was able to significantly increase the expression of WAVE3 mRNA and protein; conversely, after cell transfection with shRNA-WAVE3, which decreases the expression of WAVE3 mRNA and protein, AA manifested a weaker apoptotic ability. Moreover, the interference of WAVE3 expression is presumably responsible for the reduced cell invasiveness induced by concentrations of AA inferior to those necessary to trigger apoptotic cell death. In addition, it was revealed that WAVE3 inhibition is mediated through the PI3K/AKT signaling pathway, where the expressions of WAVE3, P53, NF-KB(P65), p-PI3K, and p-AKT were significantly inhibited while t-PI3K and t-AKT remained unchanged [41]. 

**Figure 5:**
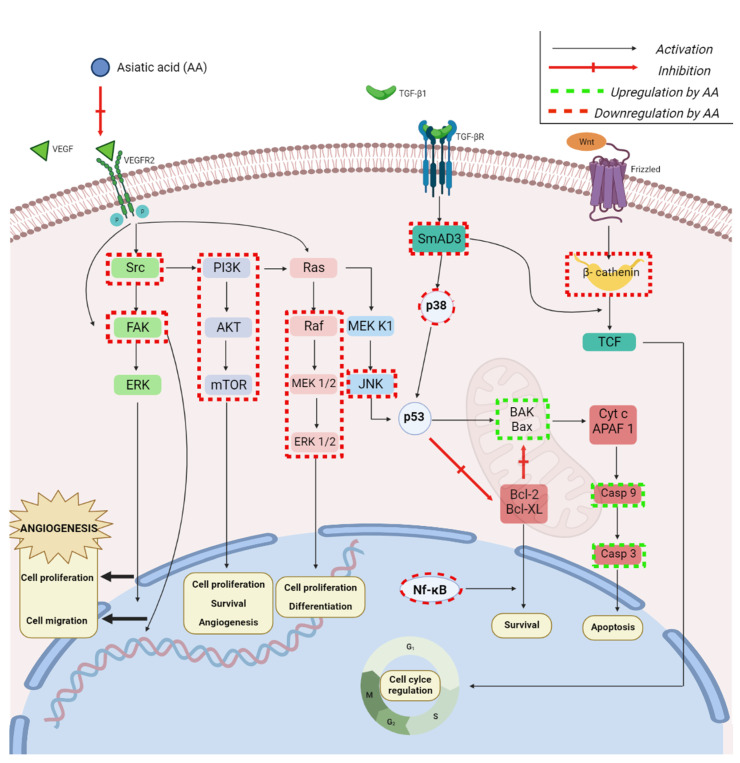
Schematic representation of the reported anticancer mechanisms of AA; key signaling pathways targeted by AA in cancer: Src/FAK/ERK signaling pathway inhibition -> inhibition of angiogenesis, cell proliferation, and migration; PI3K/Akt signaling pathway inhibition -> inhibition of angiogenesis, cell survival, and proliferation; inhibition of MEK/ERK pathway -> inhibits cell proliferation and differentiation; inhibition of TGF-β1/Smad3 signaling pathway -> inhibition of cell survival; inhibition of Wnt/β-catenin signaling pathway -> inhibition of cell proliferation and migration. Created with BioRender.com (accessed on 8 May 2022).

AA may also act as an antiangiogenic agent by inhibiting the in vitro proliferation and migration of vascular endothelial cells; these findings were validated in vivo where AA reduced breast tumor growth and prevented its spreading to the lungs by inhibiting vascular permeability [43]. At the molecular level, AA altered the VEGF/VEGFR2 signaling pathway by inhibiting VEGF expression and VEGFR2 phosphorylation, which led to the down-regulation of the Src/FAK/ERK1/2 downstream; subsequently, the compound induced tumor anti-angiogenic effects and inhibited vascular permeability thus preventing breast cancer growth and metastasis (Figure 5).

One major therapeutic challenge is the treatment of drug-resistant breast cancer; as such, AA was tested on doxorubicin-resistant MCF7 cells where it induced cell death by multiple mechanisms [44]; AA activated intrinsic apoptosis in a dose- and time-dependent manner, thus revealing the important role played by mitochondria in the response of cancer cells towards treatment. Contrary to the previous study, the authors concluded that AA particularly targets the estrogen receptor transcriptional activity, thus acting stronger against ER(+) cells. AA increased the activity of the P-gp (P-glycoprotein) energy-dependent efflux pump, which is overexpressed in chemoresistant cancers, without altering the protein expression of P-gp, and thus reversed doxorubicin resistance in breast cancer cells; it also indirectly activated the NF-κB transcription in doxorubicin-resistant breast cancer cells. In addition, AA significantly inhibited the phosphorylation of AMPKα, which is enhanced in drug-resistant breast cancer cells and which acts as an energy sensor whose activation is vital for autophagy and, subsequently, chemosensitivity; these findings were validated by the attenuation of AA cytotoxic effects through the addition of an AMPK activator and, conversely, by the prevention of its cytotoxicity as a result of AMPK knockdown. The expression of programmed cell death ligand 1 (PD-L1) was down-regulated by AA; PD-L1 was revealed as an important co-inhibitory factor of the immune reaction and plays an essential role in various neoplasia where it can reduce the host immune response towards tumor cells [45].

The ability of AA to overcome cancer drug resistance was also studied in cisplatin-resistant nasopharyngeal carcinoma [46]; the authors reported that AA acts as cytotoxic agent in a dose- and time-dependent manner by altering both intrinsic and extrinsic apoptotic pathways. Thus, the compound modified the mitochondrial membrane potential and the death receptor-initiated pathway, increased the expression of the apoptotic Bax and Bak proteins, and up-regulated the apoptotic caspases 3, 8, and 9. In addition, the authors showed that the up-regulation of caspase-9 expression was mediated by the phosphorylation of the p38 and ERK1/2 pathways, which are two of the three major MAPK cascades [47]; the phosphorylation of p38 is triggered by both mitogens and acute cellular stress and may lead to proliferation or, conversely, cell cycle arrest, depending on the activation’s duration and/or intensity (Figure 5). 

AA was found active against lung cancer through apoptosis induction in a time- and dose-dependent manner, in particular after treatment with an autophagy inhibitor [48]. As previously reported for breast cancer, AA induced the activation of caspases 3 and 9, altered the mitochondrial membrane potential, and induced reactive oxygen species generation, thus revealing mitochondria as the main target of the anticancer activity. The expression of microtubule-associated protein 1 light chain 3 (LC3), a specific marker used to monitor autophagy [49], was elevated through the conversion of the LC3-I form into LC3-II, thus indicating the induction of a protective autophagy by AA treatment, which can be reduced by autophagy inhibitors. The same lung cancer cell line, A549, was used to assess the effect of AA on the epithelial–mesenchymal transition (EMT) induced by the transforming growth factor-β1 (TGF-β1) [50]; it was found that AA inhibited both the viability and the migration ability of A549 cells treated with TGF-β1. More in-depth testing revealed that the underlying mechanism consisted in the increase in mRNA and E-cadherin expression as well as the decrease in Snail, N-cadherin, vimentin, and β-catenin expressions. Snail acts as a key inducer of the epithelial–mesenchymal transition by suppressing the expression of E-cadherin which is considered the hallmark of EMT; it strongly influences cell survival and immune regulation [51]. It was revealed that the high expression of Snail is strongly associated with tumor survival and recurrence; therefore, the inhibition of Snail by AA may pinpoint it as an effective anticancer agent. Another hallmark of the EMT is the overexpression of N-cadherin, which reveals the development of an aggressive tumor, able to metastasize to other locations within the body; therefore, N-cadherin may serve as therapeutic target aiming to prevent tumor metastasis and increase chemosensitivity [52]. Vimentin was also identified as a marker of the EMT, thus promoting metastasis [53]; β-catenin, a core component of the cadherin protein complex, is essential for the activation of the Wnt/β-catenin signaling pathway, which regulates important cellular functions including proliferation and migration [54]. The inhibition of all these EMT markers by AA makes the compound highly promising in acting as an effective treatment against tumor proliferation and migration. Another study focused on the activity of AA on the positive (Smad3) and negative (Smad7) transcription factors involved in the TGF-β1 downstream signaling [55]; AA was used as Smad7 inducer and was associated with naringenin, a Smad3 inhibitor, in order to effectively inhibit tumor growth in murine models of melanoma and lung carcinoma by inducing natural killer (NK) cells. As a result, the association of AA and naringenin produced an additive effect in inhibiting the TGF-β1/Smad3 signaling (Figure 5), thus promoting the production of NK cells and triggering a stronger immune response against cancer. The same research group investigated a second underlying mechanism in the combined therapy of AA with naringenin, consisting in the suppression of matrix metalloproteinases (MMPs) activated by TGF-β1 [56]; the investigators reported that AA enhanced the expression of Smad7 and, as a result, suppressed the TGF-β1/Smad3 signaling as well as the activation of MMP2 by altering the NF-κB-membrane-type-1 MMP (MT1-MMP) axis. Combined with naringenin, which inhibited the Smad3-mediated MMP2 transcription, AA was able to block the metastatic process by altering MMP2 transcription, activation, and function through a TGF-β1/Smad-dependent pathway.

Lung cancer can become resistant to chemotherapy and thus may evolve into poor clinical outcome; a frequently involved mechanism in multidrug resistance (MDR) is the overexpression of the previously mentioned P-glycoprotein (P-gp, MDR1), which may be the target of MDR modulators. AA was tested by Cheng et al. [57] as an MDR modulator in MDR1- overexpressing cisplatin (DDP)-resistant A549/DDP lung cancer cells where the phytocompound significantly increased the cytotoxicity of cisplatin against cisplatin-resistant cells without having any impact on the cisplatin treatment of chemosensitive A549 cells. The mechanism behind this effect resides in the suppression of the MDR1 gene transcription, which leads to a low P-gp expression and the intracellular accumulation of its substrate, Rhodamine 123. The overexpression of P-gp is presumably associated with the nuclear localization of the Y-box binding protein 1 (YB1) in certain solid tumors; its expression is mediated by several signaling pathways, in particular NF-kB and MAPK. In addition, the MAPK pathway regulates YB1 phosphorylation [58]. AA inhibited YB1 nuclear translocation in the cancer cells, while treatment with cisplatin alone did not induce this phenomenon. The transcription factor NF-κB is essential in multiple immune and inflammatory reactions; the NF-κB signaling pathway involves five transcriptional monomers that remain inactive in the absence of pathological stimuli. When such stimulation occurs, it is immediately followed by the degradation of IkB proteins and activation of NF-κB, the most abundant form being the p65: p50 heterodimer; subsequently, the transactivation of several effector molecules takes place and facilitates the pathogenesis of numerous diseases [59]. AA inhibits the degradation of IkB proteins and, subsequently, the NF-kB/p65 nuclear translocation, finally resulting in blocking the NF-kB/p65 activation. The mitogen-activated protein kinase (MAPK) family is essentially involved in signaling cascades, which mediates the transmission of extracellular stimuli to intracellular targets [60]; AA inhibits the phosphorylation of ERK1/2, p38 and JNK thus inhibiting three out of the four defined MAPK cascades, p38, and JNK MAPK pathways being associated with cell apoptosis and ERK1/2 with cell proliferation and differentiation (Figure 5). In particular, the overexpression of ERK pathway components was revealed as a specific marker for cancer, inflammation, and other chronic disorders. Collectively, AA inhibited P-gp expression in A549/DDP cells through the down-regulation of YB1, which in turn was mediated through the NF-kB and MAPK pathways.

Since mitochondria was revealed as potential target for AA, the phytocompound can be considered as mitocan (mitochondria targeting anticancer agents); Swargiary et al. performed the molecular docking of sixty phytocompounds against the glycolytic enzyme hexokinase II (HK2), which is commonly overexpressed in numerous types of cancer and facilitates ATP generation as well as protection against apoptosis [61]. The authors identified AA as potential natural mitocan; these findings were previously suggested by Kahnt et al., who designed the bioconjugate of AA with rhodamine B that was cytotoxic on several cancer cell lines in nanomolar concentrations.

AA inhibited the proliferation and migration of colon cancer cells in a time- and dose-dependent manner [62] by delaying G2/M and S phase progression; the results indicated the down-regulation of several phosphorylated proteins such as PI3K, Akt, mTOR, and ribosomal protein S6 kinase as well as the up-regulation of the Pdcd4 expression. In a similar manner as with lung cancer, AA down-regulated the expression of epithelial–mesenchymal transition markers in a dose-dependent manner; collectively, AA induced the apoptosis of colon cancer cells and prevented their migration by regulating the expression of Pdcd4 through the PI3K/Akt/mTOR/p70S6K signaling pathway (Figure 5).

The activity of AA on tongue cancer cells was investigated by Li et al. [63], who reported its apoptotic in vitro and in vivo effects mediated through several underlying mechanisms. The application of AA in Tca8113 cancer cells led to the increase in calcium levels as well as the level of calcium-dependent protease calpain; calpains are cysteine proteases that act on various substrates and participate in the regulation of cell migration and cell cycle progression [64]. Calpains are presumably activated by increased intracellular calcium levels and may induce the cleavage of pro-caspase 12 to caspase 12, which triggers endoplasmic reticulum (ER)-mediated apoptosis. In addition, the ER stress induced by AA promotes the phosphorylation of IRE1α and JNK through the intervention of glucose-regulated protein 78 (GRP78), which is an ER stress sensor that enhances cell survival [65]. Overall, AA activated the ER Grp78/IRE1α/JNK signaling pathway, which resulted in the activation of mitochondrial apoptosis as revealed by the suppression of Bcl-2 and enhancement of Bax expressions; subsequently, cytochrome C was released in the cytosol and activated caspase-3, further inducing apoptotic cell death.

### 2.2. Antidiabetic Activity

The crude extract of *Centella asiatica* was tested against rat kidney and brain oxidative and inflammatory stress caused by diabetes [66]; the authors reported elevated levels of malondialdehyde (MDA), TNF-α, and IFN-γ and reduced antioxidant status in both kidney and brain of diabetes-induced rats. Treatment with *C. asiatica* extract containing AA as one main component significantly reduced the levels of MDA, TNF-α, and IFN-γ but simultaneously increased the levels of IL-4 and IL-10. In diabetes, cell malfunction is induced by excessive production of ROS accompanied by an altered lipid metabolism, which finally leads to MDA, a highly toxic by-product able to cause DNA damage that can be used as marker for oxidative stress [67]. TNF-α is an adipocytokine involved in the development of insulin resistance [68]; in addition, interferon- γ (IFN-γ) contributes to diabetes pathogenesis through the increased expression of major histocompatibility complex (MHC) class I and class II proteins as well as other adhesion molecules on pancreatic β islets cells [69]. Furthermore, the Th1/Treg cells imbalance in diabetic patients triggers low interleukin-10 production, which is a potent anti-inflammatory cytokine that inhibits TNF-α production. Interleukin-4 (IL4) is a Th2 cytokine that plays a well-known role in the immune system and, simultaneously, its interaction with specific receptors, IL4R, strongly promotes pro-metastatic behavior of epithelial cells (i.e., proliferation, migration, survival) which exhibit overexpressed IL4 receptors [70]. Therefore, by inhibiting pro-diabetes and stimulating anti-diabetes factors, AA acts as an efficient tool against diabetes-related kidney and brain stress, thus preventing diabetes complications.

The protective effect of AA against type-II diabetes was investigated by Sun et al., who reported its ability to regulate the PI3K/AKT/GSK-3β signaling pathway in a diabetes murine model [71]. The glycogen synthase kinase-3β (GSK-3β) is a serine/threonine protein kinase involved in the molecular pathophysiology of many disorders, which may interact with several mitochondrial proteins, including PI3K-Akt, thus interfering with multiple mitochondrial processes [72]. In addition, GSK-3β regulates the expression of glucose-6-phosphatase (G6P) and, together, the two enzymes regulate glycogen synthesis and gluconeogenesis leading to the phosphorylation of the insulin receptor substrate-1, finally resulting in insulin resistance [73]. The administration of AA down-regulates the expressions of PI3K, AKT, insulin receptor, and insulin receptor substrate-1 mRNA and further decreases the GSK-3β and G6P mRNA expressions, therefore ameliorating glucose and lipid levels as well as glycogen synthesis in diabetic mice.

Diabetes frequently induces related nephropathies; since AA is a well-documented anti-diabetic agent, an interesting aspect would be its potential protective effect on podocytes as well as the underlying mechanism. Chen et al. reported that AA effectively reduce urinary albumin and counteracts the abnormal morphological changes of podocytes in a dose-related manner [74]; in addition, the authors revealed the up-regulation of nephrin and down-regulation of desmin. Mechanistically, AA reduced the phosphorylation of JNK protein, thus suppressing the activation of the JNK signaling pathway and inhibiting the production of ROS.

### 2.3. Antiinflammatory Activity

Inflammation can be defined as the immune response of the organism, induced by pathogens, altered cells, or toxic drugs, which may trigger acute or chronic reactions in most organs; multiple inflammatory signaling pathways were identified, particularly NF-κB, MAPK, and JAK-STAT pathways [75]. The nuclear factor-κB (NF-κB) pathway involves numerous transcription factors such as IL-1β, IL-6, tumor necrosis factor-α (TNF-α), and monocyte chemotactic protein 1 (MCP-1), which are increased in pelvic inflammatory disease [76]; it was reported that AA inhibits the activation of the NF-κB pathway and also of the nucleotide-binding domain-like receptor protein 3 (NLRP3) inflammasome, which mediates the release and maturation of IL-1β, thus significantly reducing the concentration of inflammatory cytokines and chemokines and ameliorating oxidative stress. The inhibition of IL-1β together with IL-6, alanine aminotransferase, and blood urea nitrogen by AA was also reported in sepsis, another inflammatory disease in which the Notch signaling pathway plays an essential role [77]; as a result, AA was able to diminish sepsis-associated liver, lung, and kidney damage, thus improving survival in experimental animals. The underlying mechanism consists in the up-regulation of the Notch3 receptor as well as the delta-like ligand (DLL4), both components of the Notch signaling pathway; furthermore, AA inhibits the binding of Notch3 to the promoter region of IL-6, thus altering the regulation of the IL-6 mRNA expression and refraining cytokine production; in addition, AA participated in the regulation of mitochondrial function.

Due to its anti-inflammatory and antioxidant properties, AA is able to act against both idiopathic and drug-induced pulmonary fibrosis by inhibiting lung injury as well as disease progression [78]. In a murine model of bleomycin-induced pulmonary fibrosis, Dong et al. reported that AA reduced the infiltration of inflammatory cells into the bronchoalveolar lavage fluid and the expression of inflammatory cytokines (i.e., IL-1β, IL-6, IL-18, and TNF-α) in lungs. In terms of molecular mechanisms, AA acted as a suppressor of TGF-β1, collagen I and III, αSMA, and matrix metalloproteinase (TIMP)-1 and, subsequently, as an inactivator of Smads and ERK1/2 signaling pathways, which have been proven essential for the differentiation of fibroblasts to myofibroblasts induced by TGF-β1; in addition, AA diminished the expression of the NLRP3 inflammasome, which was reported to associate with fibrosis development. The NLRP3 inflammasome is activated as a result of the interaction between the intracellular sensor NLRP3 (NOD-, LRR-, and pyrin domain-containing protein 3) and various stimuli and triggers the release of pro-inflammatory cytokines (i.e., IL-1β and IL-18) [79]; therefore, its inactivation by AA induces anti-inflammatory effects. Pulmonary fibrosis is the most common complication of the autoimmune systemic sclerosis leading to high rates of mortality; the administration of AA in murine models of scleroderma led to the attenuation of the histopathological progression in lungs as well as of the conversion of fibroblasts in muscle fibroblasts [80]. The underlying mechanisms consisted in the down-regulation of E-selectin and the decrease in the serum levels of anti-DNA topoisomerase-1 autoantibody; in addition, AA normalized the expression of TGF-β1 and the ratio of phosphorylated-Smad2/3/Smad2/3. Thus, the authors concluded that AA targets the TGF-β1/Smad2/3 signaling pathway by inhibiting the activation of Smad2/3 through the down-regulation of the Smad2/3 phosphorylation process.

AA was found to be useful as well in the treatment of osteoarthritis, which evolves through the progressive degradation of the articular cartilages presumably due to chondrocyte hypertrophy and fibrosis. AA significantly ameliorated both chondrocyte hypertrophy and fibrosis without altering the chondrogenic phenotype by activating the AMP-activated protein kinase (AMPK) which, in turn, inhibited the PI3K/AKT signaling pathway associated with hypertrophy and fibrosis in various tissues [81]. Osteoarthritis is related to other conditions such as obesity due to the direct link between the aberrant lipid metabolism and inflammatory processes combined with chondrocytes’ excessive catabolism [82]. 

AA can combat the inflammation induced by palmitate acid in human chondrocytes and also prevents the degradation of the extracellular matrix in vivo. The authors revealed that the anti-inflammatory and chondrocyte protective effects were caused by the inhibition of the NF-κB pathway mediated through the binding of AA to the MD-2/TLR4 complex, as highlighted by molecular docking. The in vitro findings were validated in vivo where AA reduced the production of proinflammatory cytokines in obese mice [82].

### 2.4. Neuroprotective Activity

**Figure 6:**
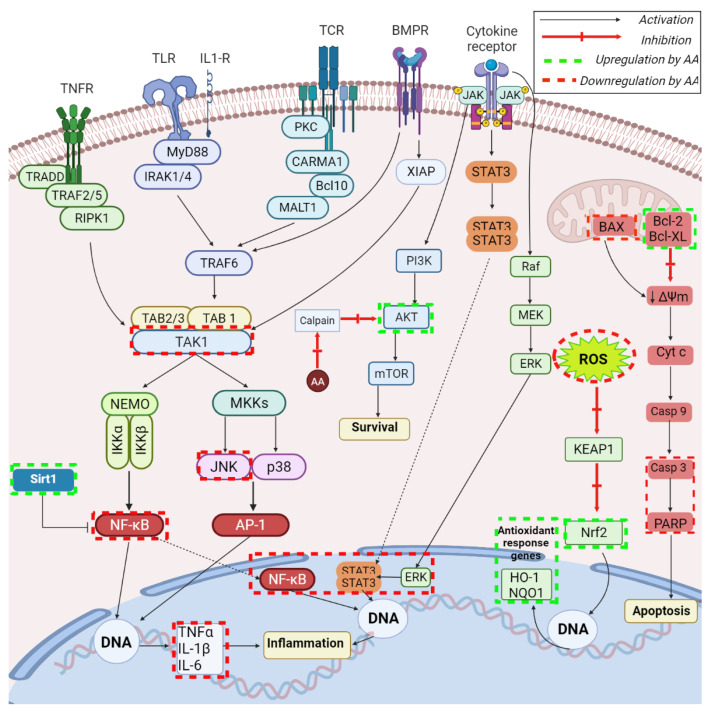
Schematic representation of the reported neuroprotective mechanisms of AA; key signaling pathways targeted by AA in neurodegeneration: inhibition of NF-kB/STAT3/ERK signaling pathway-> down-regulation of TNF-α, IL-1β and IL-6; up-regulation of Bcl and down-regulation of Bax -> anti-inflammatory and antiapoptotic effect; inhibition of TAK1-JNK pathway -> anti-inflammatory effect; up-regulation of Nrf2/HO-1 pathway -> reduction of ROS -> antioxidant effect. Created with BioRender.com (accessed on 8 May 2022).

AA has exhibited neuroprotective effects in various conditions, either neurotoxic or neurodegenerative. As a result of illegal drug administration, such as methamphetamine, neuroinflammatory processes may be triggered in the brain through the induction of the TNF receptor expression as well as the production of pro-inflammatory cytokines such as TNFα and IL-6 (Figure 6) [83]. AA was able to significantly inhibit the methamphetamine-induced expression of TNFR in a concentration-dependent manner, and the production of TNFα and IL-6 in neuronal cells. Further investigations revealed that the phytocompound inhibited the nuclear translocation of NF-κB/STAT3 and the phosphorylation of ERK, thus exerting its protective effect by inhibiting the NF-kB/STAT3/ERK signaling pathway, which acts as a regulatory mechanism for TNFα and IL-6; in addition, the neuronal mitochondrial apoptotic processes were dramatically suppressed, as revealed by the inhibition of caspase-3 and PARP fragmentation, decreased levels of the pro-apoptotic Bax protein, and increased concentrations of the anti-apoptotic Bcl-xL protein (Figure 6). 

In terms of protection against neurodegenerative diseases, AA showed efficiency as an anti-inflammatory agent in Parkinson’s disease using 1-methyl-4-phenyl-pyridine (MPP+)-induced SH-SY5Y cells as in vitro model [84]. The phytocompound was able to suppress the activation of the NLRP3 inflammasome in BV2 microglia cells by down-regulating the mitochondrial reactive oxygen species thus protecting the dopaminergic neurons; NLRP3 inflammasome plays an essential role in the inflammatory process that triggers Parkinson’s disease development. In addition, AA protected the BV2 microglia cells against lipopolysaccharide (LPS)-induced toxic effects and increased their cell viability while also diminishing the MPP+-induced mitochondrial dysfunction in SH-SY5Y cells. A similar study was conducted by Qian et al. on LPS-induced neuroinflammation in BV2 microglia cells where the investigators reported a significant attenuation of nitric oxide production through the concentration-dependent inhibition of the nitric oxide synthase expression [85]. Subsequently, AA inhibited the secretion of inflammatory cytokines, such as TNF-α, IL-1β, and IL-6; furthermore, it was shown that AA regulates the Sirt1/NF-κB signaling pathway, as revealed by its ability to increase Sirt1 expression, which in turn reduces the NF-κB p65 acetylation and finally results in the NF-κB inactivation (Figure 6). Therefore, AA could serve as treatment for neuroinflammatory disorders that evolve through microglial activation. The Parkinson’s disease pathogenesis was also linked to alterations of the mitochondrial complex I, which normally facilitates the association of α-synuclein with mitochondria [86]; as a result, one can notice the overexpression of α-synuclein, which causes the generation of oxidant radical within the mitochondria followed by structural and functional mitochondrial aberrations. Through its antioxidant effects, AA counteracts oxidative stress and apoptosis in mitochondria while maintaining membrane integrity and ATP production; at the molecular level, AA inhibits the translocation of α-synuclein into mitochondria. Collectively, considering the essential role of mitochondria in the development and progress of Parkinson’s disease, the authors highlighted AA’s potential to act as prophylactic and therapeutic agent against the disease.

A key mechanism of antiepileptic drugs consists in the inhibition of the glutamate release, which results in the reduction of neuronal excitability; the application of AA in hippocampal synaptosomes in rats induced the inhibition of glutamate release in a concentration-dependent manner [87]. In-depth investigations revealed that the inhibitory effect of AA depended on the extracellular calcium, and also inhibited the phosphorylation of protein kinase C; these findings were validated by the reported suppression of AA’s activity by ω-conotoxin MVIIC, a N- and P/Q-type calcium channel inhibitor, or GF109203X, a protein kinase C inhibitor. Collectively, AA significantly reduced the calcium influx through the activation of N- and P/Q-type calcium channels, resulting in the suppression of protein kinase C activity and finally leading to the decrease in glutamate release with beneficial outcomes in epileptic patients. Remaining in the same sphere of pathology, epilepsy can be associated with cognitive impairment, which may also occur as adverse effects of conventional antiepileptic drugs; based on the previously reported antiepileptic activity of AA, Lu et al. investigated its effects on cognitive deficits in rats exposed to kainic acid-induced seizures [88]. AA was able to attenuate seizure, memory deficits, and neuronal damage through inactivating calpain, activating protein kinase B (AKT), and ameliorating mitochondrial alteration at the hippocampal level. Further studies showed that AA prevented the alteration of proteins involved in mitochondrial functions such as lipoamide dehydrogenase, glutamate dehydrogenase 1, ATP synthase, and mitochondrial deacetylase sirtuin-3 (SIRT3). Cognitive impairment can also be associated with certain anticancer drugs such as 5-fluorouracil that induce intracellular oxidative stress; AA may interfere with the hippocampal neurogenesis and memory [89]. Thus, AA reversed the effects of fluorouracil and prevented the decrease in Notch1, SOX2, nestin, DCX, and Nrf2, all factors involved in cell functions and self-renewal as well as in neurogenesis and reduction-oxidation homeostasis, while decreasing p21 (a negative regulator of cell cycle) positive cells and malondialdehyde (MDA, marker of oxidative stress) levels. Therefore, one can state that the phytocompound prevented the down-regulation of neurogenesis and ameliorated the memory impairments induced by the chemotherapeutic drug by diminishing the oxidative stress and providing a neuroprotective effect. In another study published by Wang et al., it was revealed that AA attenuated kainic acid-induced seizures in mice by reducing the release of inflammatory interleukin (IL)-1beta, IL-6, tumor necrosis factor-alpha and prostaglandin E2, as well as diminishing the activity of cyclooxygenase-2 activity and NF-κB p50/65 in the hippocampus; in addition, AA lowered the generation of ROS and enhanced the activity of glutamine synthetase, consequently increasing the hippocampal glutamine level. AA may serve as nutraceutical agent against seizures due to its beneficial effects on cell viability in nerve growth factor (NGF)-treated PC12 cells where the phytocompound diminished the release of calcium ions [90].

Moving outside of the hippocampal area, Wong et al. investigated the effects of *Centella asiatica* extracts on the α-amino-3-hydroxy-5-methyl-4-isoxazolepropionic acid receptors (AMPARs), which are widely distributed throughout the central nervous system; they are ionotropic glutamate receptors that mediate rapid excitatory neurotransmission and that presumably represent the cellular mechanism involved in learning abilities and memory. The extract of *C. asiatica* clearly enhances the surface expression of AMPARs in the entorhinal cortex of rat brains, thus increasing the amplitude of the spontaneous excitatory postsynaptic currents; one may conclude that the vegetal extract containing mainly AA and asiaticoside is responsible for triggering an increased response of AMPARs at postsynaptic level and, subsequently, producing cognitive enhancements. Moreover, the authors revealed that only small doses of extract manage to modulate AMPAR-mediated current responses, thus suggesting a synergistic interaction between components; the optimal combination between *C. asiatica* extract is presumably analogous to the nerve growth factor [91]. Controversially, the comparative study of three types of *C. asiatica* extract (raw, enriched, and depleted of triterpenes) was tested in terms of antioxidant and cholinergic modulatory effects, which were reported as the main mechanisms of action involved in the extract’s efficacy against memory disorders [92]; the raw methanolic extract was revealed as the most efficient antioxidant and anti-amnesis agent. However, it should be emphasized that the triterpene-free extract induced stronger antioxidant effects than the triterpene-enriched extract, combined with a comparable anti-amnesic effect, which strongly suggested that triterpenes are not the only active principles in the *C. asiatica* extract and that synergistic and/or antagonistic interactions between components should really be considered for future studies.

AA was revealed as a useful option in the prevention of hypoxic ischemia-induced injuries, which may result in later seizures, impaired learning, cerebral palsy, and epilepsy by targeting apoptosis pathways [93]. One such apoptotic pathway is the TAK1-JNK pathway, which involves the activation of TGF-β-activated kinase-1 (TAK1) by various stimuli, which in turn activates the c-Jun N-terminal kinase (JNK) and p38 MAPK stress factor-associated pathways; therefore, TAK1 is a key player in cell death regulation (Figure 6). Wang et al. showed that the hypoxia-mediated activation of TAK1 can be suppressed by AA, thus resulting in the alleviation of hypoxia-induced neuronal apoptosis; these findings were validated by the use of a TAK1 inhibitor, which also suppressed the hypoxia-mediated up-regulation of the expressions of p-JNK, caspase3, p53, and p-c-Jun in brain cortex in a similar manner with AA. Another pathology where AA proved beneficial is traumatic brain injury, which may occur in individual of all ages and health status and may lead to death or disability [94]); the phytocompound is able to improve neurological dysfunction and to reduce brain edema and neuronal apoptosis while also fighting oxidative stress. The underlying mechanism consists in the up-regulation of regulator nuclear factor erythroid 2-related factor (Nrf2), which acts as cytoprotective and regulates in turn gene expression for coding of heme oxygenase-1 (HO-1)–also a cytoprotective agent; therefore, the authors attributed AA with the ability to target the Nrf2/HO-1 pathway, which is crucially involved in oxidative stress (Figure 6).

The neuroprotective effects of AA were also tested in a rat model of glaucoma where the phytocompound was intravitreally administered followed by the assessment of densities and function of retinal ganglion cells [95]. The study reported the amelioration of retinal dysfunction through the up-regulation of antiapoptotic Bcl-2 protein and down-regulation of pro-apoptotic Bax protein and caspase-3 (Figure 6), therefore indicating AA as neuroprotective and anti-glaucoma agent.

### 2.5. Cardioprotective Activity

**Figure 7:**
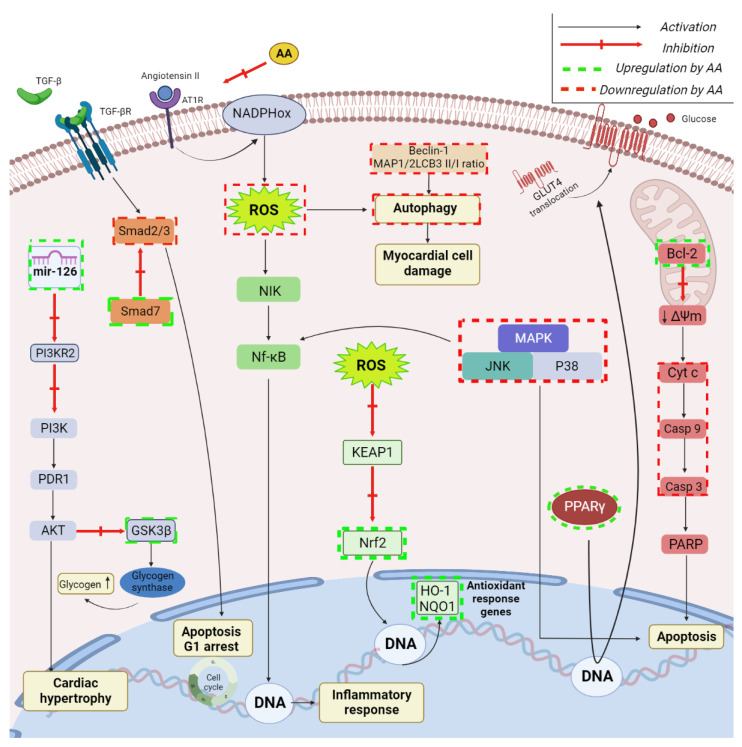
Schematic representation of the reported cardioprotective mechanisms of AA; key signaling pathways targeted by AA in neurodegeneration: MAPK/mitochondria-dependent apoptotic pathway inhibition -> antioxidant effect; Akt/GSK-3β-mir-126 mediated signaling pathway activation -> reduces myocardial hypertrophy; TGF-β1/Smad2/3 phosphorylation inhibition -> reduces myocardial hypertrophy; increasing the expression of Nrf2, HO-1, and NQO-1 -> antioxidant effect; Ang II-AT1R-NADPH oxidase-NF-κB pathway inhibition -> anti-inflammatory effect. Created with BioRender.com (accessed on 8 May 2022).

AA exerts cardioprotective activity through various mechanisms, one of them being the antioxidant effect; thus, AA was able to decrease the levels of ROS and malondialdehyde in a mouse model of myocardial ischemia-reperfusion injury (MIRI), which stands as a major cause of heart failure in patients with coronary pathologies [96]. MIRI was associated with abnormal generation of ROS, which in turn triggered the activation of autophagy, thus resulting in myocardial cell damage; the intervention of AA induced an improved cardiac function as a result of decreased autophagy. In-depth studies showed that the ROS-mediated autophagy was prevented by AA through the inhibition of p38 phosphorylation and the up-regulation of Bcl-2 accompanied by a decreased expression of autophagy markers such as beclin-1 and microtubule-associated proteins 1A/1B light chain 3B II/I ratio (Figure 7). Furthermore, very recent studies of the same group reported that AA reduces ROS generation through the inhibition of MAPK/mitochondria-dependent apoptotic pathway, as revealed by a series of events such as limited phosphorylation of p38-MAPK and JNK-MAPK, balanced Bcl-2/Bax ratio, reduced cytochrome c release, caspase cascade inhibition, and reduced apoptosis (Figure 7) [97]. MIRI is also associated with the suppression of oxidative metabolism in myocardial cells in favor of glycolysis as ATP generator, which simultaneously leads to the accumulation of lactate and hydrogen ions, thus impairing calcium ions uptake by the sarcoplasmic reticulum and the antioxidant mechanisms, finally resulting in cell apoptosis and necrosis [98]. AA is able to modulate the glycometabolism by reducing LDH and CK activities, and to suppress the apoptotic process via the activation of the Akt/GSK-3β signal pathway in ischemic cardiomyocytes; in addition, the phytocompound inhibited glycogen breakdown, up-regulated the glucose regulator PPARγ expression at mRNA as well as protein level and also facilitated GLUT4 translocation to plasma membrane, thus inducing optimized glucose utilization (Figure 7). The revealed underlying mechanism involving the Akt-dependent cellular events was validated through the administration of an Akt inhibitor, which reversed the effects of AA.

An independent risk factor of several cardiovascular pathologies is the cardiac hypertrophy, which was hypothesized to involve microRNA-126 (miR-126) whose role in angiogenesis was already revealed [99]. The authors had previously established the protective role of AA against cardiac hypertrophy during the investigation of AA’s involvement in the treatment of overpressure-induced cardiac fibrosis in hypertensive rats [100]. The study revealed that AA was able to reduce systolic blood pressure and myocardial hypertrophy and fibrosis; the underlying mechanism consisted in the inhibition of TGF-β1/Smad2/3 phosphorylation accompanied by the increase in the Smad7 expression (Figure 7). Additionally, the phytocompound exerted antioxidant effects while increasing the expression of Nrf2, HO-1, and NQO-1 both in vivo and in vitro; these findings were validated by the administration of siRNA for Smad7 or Nrf2, which partially reversed the inhibitory effects of AA against the AngII-induced cardiac fibrosis. Following these studies, the same research group has deepened their investigation aiming to establish the mechanistic regulatory effect of miR-126 in cardiac hypertrophy as well as its participation in AA’s anti-hypertrophy effect. AA up-regulated the expression of miRNA-126, which is markedly down-regulated in an AngII-induced model of cardiac hypertrophy; the study revealed that miR-126 directly targets PIK3R2, as shown by the inverse relationship between the concentrations of miR-126 and PIK3R2. The up-regulation of miR-126 by AA induces a reduction of PIK3R2, which in turn triggers the inhibition of the PI3K/Akt signaling pathway, thus providing cardio-protection (Figure 7); overall, AA may act as potential preventive agent against cardiac hypertrophy and fibrosis.

Atherosclerosis can be defined as a chronic inflammatory process, which results in cholesterol accumulation in plaques within the arterial vessels, gradually restricting blood flow; the pathophysiological process begins with endothelium dysfunctions mediated by intra- and intercellular events triggered by proatherogenic stimuli [101]. AA can act as protective agent for the endothelial barrier in human aortic cells by inhibiting the effects of tumor necrosis factor (TNF)-α, thus stabilizing F-actin and diphospho-MLC and preventing the structural rearrangement of vascular endothelial cadherin and β-catenin [102]; therefore, AA can be potentially used as preventive agent in early atherosclerosis.

The antioxidant and anti-inflammatory activities of AA can also be exploited against hemodynamic alterations (i.e., high blood pressure, heart rate, vascular resistance) caused by disruption of the renin-angiotensin system [103]. AA alleviated RAS activation, subsequently decreasing plasma angiotensin II, the activity of angiotensin-converting enzyme and the expression of AT1R protein, while increasing the expression of AT2R protein; in addition, AA counteracted oxidative stress and inflammation by reducing plasma levels of TNF-α, NF-κB, and phospho-NF-κB protein, thus inhibiting the Ang II-AT1R-NADPH oxidase-NF-κB pathway (Figure 7) and collectively acting as an ACE inhibitor such as captopril.

### 2.6. Hepatoprotective Activity

AA acts as protective agent against liver injuries presumably due to its antioxidant effects mediated through the direct targeting of mitochondria. Lu et al. investigated the preventive effect of the phytocompound on ischemia/reperfusion-induced liver damages and reported decreased levels of aminotransferase, TNF-α, and malondialdehyde and increased concentrations of GSH accompanied by intensified activities of hepatic SOD and catalase; collectively, these findings indicate the antioxidant activity of AA in liver tissues and an overall improvement of the mitochondrial respiration [104]. In addition, when tested on liver mitochondria from normal rats, AA ameliorated the respiratory dysfunction induced by the anoxia/reoxygenation process and inhibited the generation of mitochondrial ROS. The suppression of ROS generation as well as apoptosis and cytotoxicity by AA was also reported by Qi et al., who revealed that the phytocompound activates the (Nrf2) signal thus triggering the up-regulation of antioxidant genes and antioxidant response element (ARE). The Nrf2 activation by AA is mediated through the Akt and ERK signaling as indicated by the administration of Akt and ERK inhibitors that not only inhibits its antioxidant activity but also decreases its cytoprotective effect [105]. Another research group reported that the protective activity of AA against ischemia/reperfusion-induced liver damages is mediated by the increased expression of PPARγ; the finding was validated through the PPARγ pharmacological inhibition, which counteracted AA’s hepatoprotective effects including the inhibition of NLRP3 inflammasome activation [106]. In vitro studies showed that AA inhibited the activation of NLRP3 inflammasome in a concentration-dependent manner, its activity being suppressed either pharmacologically or by PPARγ genetic knockdown; subsequently, the authors revealed an inhibitory effect of the phytocompound on ROS production and phosphorylation processes of several kinases such as JNK and p38 MAPK. The antioxidant and anti-inflammatory activity of AA can also be exploited against fulminant hepatic failure where the phytocompound exerts its therapeutic activity through MAPK inhibition and NF-κB activation, a mechanism that involves the up-regulation of Nrf2 via the activation of the AMPK/GSK3β pathway [107].

AA has also proven its therapeutic effect against acute liver injuries, as revealed by decreased serum levels of alanine aminotransferase (ALT) and aspartate transaminase (AST) in mice; at the cellular level, the phytocompound inhibited apoptosis and induced cell cycle arrest in S/G1 phase [108]. Mechanistically, AA suppressed the activation of the PERK/ATF6 and IRE1 pathway involved in apoptosis initiation, which in turn alleviated the extent of the endoplasmic reticulum stress (ERS); since ERS is also linked to autophagy, the process was significantly induced by AA as revealed by cell morphological changes as well as increased expressions of LC3II/I, Beclin-1, Atg5, and Atg7. The ERS also participates in the development of non-alcoholic fatty liver disease, which can be ameliorated by AA and significantly alleviates lipidosis both in vitro and in vivo, thus counteracting hepatocyte damage and lipid metabolic alterations [109]. Further investigations emphasized the regulatory effect of AA on key factors involved in lipid metabolism such as sterol regulatory element-binding protein 1c (SREBP-1c), encoding carboxylase, liver X Receptor Rα (LXRα), fatty acid synthase (FAS), and AMP-activated protein kinase (AMPK), which finally results in decreased lipogenesis in hepatocytes. In addition, AA inhibits the NF-kB pathway, thus triggering an anti-inflammatory effect and reducing the ERS, thereby ameliorating liver lipid deposition.

Liver fibrosis that emerges as a healing response to liver chronic damage of various causes currently benefits of very few therapeutic options; therefore, the search for effective treatment is ongoing in order to prevent further development of liver cirrhosis. AA exhibited protective activity against experimental models of liver fibrosis by acting as regulatory factor on the PI3K/AKT/mTOR and Bcl-2/Bax signaling pathways; these mechanisms induced a clear prevention of the progression of liver fibrosis, as revealed by the assessment of liver fibrosis-related indexes (i.e., body weight, biochemical parameters, biomarkers, histological alterations, etc.) [110]. The investigations conducted by the same research group also revealed that AA increased the expression of nuclear Nrf2, which in turn increased the levels of HO-1, NQO-1, and GCLC proteins; simultaneously, it decreased the concentration of nuclear NF-κB which deactivated the NF-κB/IκBα signaling pathway. The phytocompound also decreased the phosphorylation process of JAK1 and STAT3, thus acting as a regulating agent on the JAK1/STAT3 signaling pathway [111]. Collectively, AA may serve as a potential antifibrosis agent against liver damage; as a result, efforts have been made to improve its biopharmaceutical properties such as the oral bioavailability and targeting efficiency by the synthesis of nanostructured lipid carriers, which significantly improved the phytocompound’s in vivo efficacy [112].

### 2.7. Other Biological Activities

Bones are dynamic tissues whose homeostasis is maintained by an equilibrium between osteoclast-mediated resorption and neoformation induced by osteoblasts strictly controlled by local and systemic factors [113]. AA inhibited osteoclast differentiation and the subsequent bone loss by inhibiting the signaling pathways mediated by the receptor activator of NF-κB ligand (RANKL) such as NF-κB and NFATc1 signalings [114]. Further in vivo studies revealed that AA counteracts bone loss in ovariectomized mice where the osteoporosis is induced by lack of estrogens; histological analysis emphasized the significant reduction of the osteoclast number while blood analysis identified low levels of TRAcP and CTX-1. Through its inhibitory activity of the osteoclast formation, the phytocompound may act as therapeutic agent against overexpressed RANKL-related osteolytic conditions.

AA was found to alleviate renal fibrosis, which occurs in various types of progressive renal diseases. Thus, unilateral ureteral occlusion surgery was conducted on rat and mouse models that developed renal dysfunction and fibrosis combined with oxidative stress and the activation of specific pathways in kidneys such as the TGF-β/Smad and Wnt/β-catenin signaling pathways [115]; AA was able to attenuate all these pathological changes, its activity being suppressed by the co-administration of a selective PPAR-γ antagonist. However, AA did not act directly on the PPAR-γ kidney levels but on the plasma levels of its endogenous ligand, 15d-PGJ2; in addition, the phytocompound induced the up-regulation of nuclear-localized SREBP-1 (nSREBP-1), which can be inhibited by fatostatin, a compound that also decreases the 15d-PGJ2 level.

The kidney protection induced by AA is also manifested against cyclophosphamide-induced cystitis as revealed by Wrobel et al. who administered the drug intraperitoneally to rats simultaneously with the oral administration of AA [116]. The combination of cyclophosphamide and AA significantly improved bladder parameters in comparison to the drug alone, additionally decreasing urothelium thickness and bladder oedema [117]. In terms of molecular mechanisms, AA contributed to the normalization of the urothelium and detrusor muscle biomarker concentrations such as malondialdehyde, TNF-α, IL-1β, and IL-6, thus acting as a protective agent against drug-induced cystitis. The anticancer drug cisplatin also triggers nephrotoxic side effects, presumably through the apoptosis of tubular epithelial cells accompanied by renal inflammation. The antiapoptotic and anti-inflammatory effects of AA were tested on cisplatin-treated mice where the phytocompound significantly reduced the level of serum creatinine and blood urea nitrogen and alleviated histological alterations. The underlying mechanism involved the up-regulation of the anti-apoptotic survivin and tubular proliferation, which led to larger numbers of proliferating cell nuclear antigen-positive cells; in addition, AA down-regulated the kidney injury molecule-1 and suppressed the production of pro-inflammatory cytokines and caspase-1 through NF-κB activation inhibition, presumably due to the Smad7 up-regulation.

## 3. Oleanolic Acid

**Figure 8:**
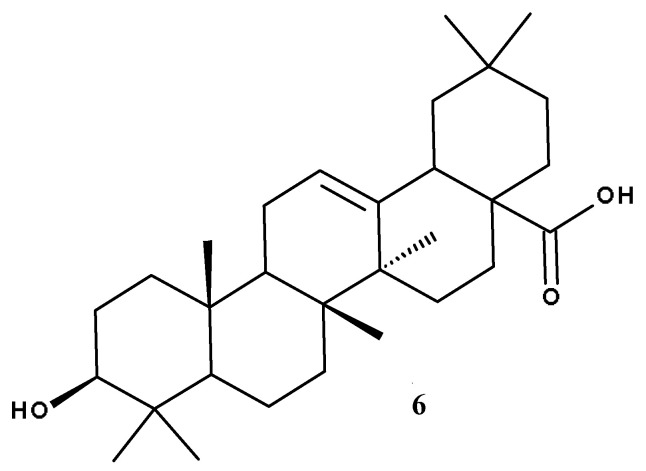
The chemical structure of oleanolic acid (**6**).

Oleanolic acid (OA, Figure 8) is a biologically active compound belonging to the large class of pentacyclic triterpenes, prevalently being found in olives and other species from Oleaceae family, often in combination with its isomer, UA [118]. In nature, OA can be found either in its free acid form, or as the corresponding saponin, linked with several sugar chains; furthermore, OA possesses numerous pharmacological properties, exerting anticancer, antiviral, antidiabetic [119], anti-inflammatory, antioxidant, cardioprotective, hepatoprotective, and anti-osteoporotic effects [120].

**Figure 9:**
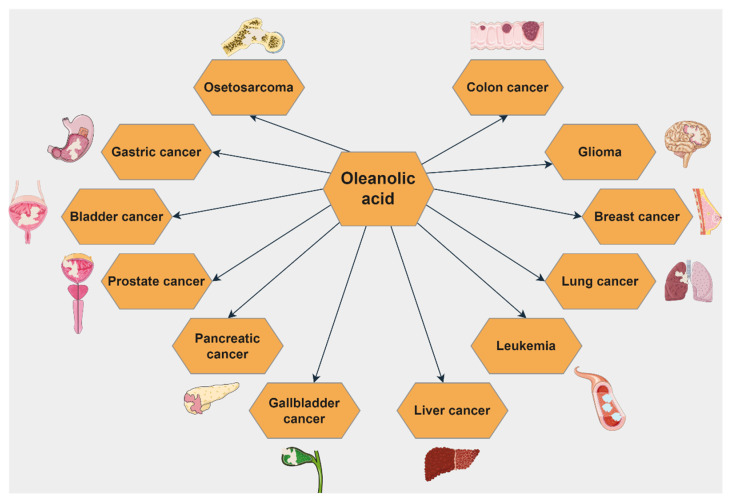
Anticancer effect of oleanolic acid in various types of cancer.

An elaborate review was published in 2016 regarding the molecular mechanisms of OA antineoplastic activity in different types of cancer (Figure 9) [121]. Another recently published review by Castellano et al. [122] depicts the molecular mechanisms involved in the antihypertensive, antihyperglycemic, and anti-inflammatory activity of OA. 

Even though the main OA pharmacological activities have been extensively studied for a rather long time, a plethora of new potential utilizations are currently under investigation.

### 3.1. Anticancer Activity

**Figure 10:**
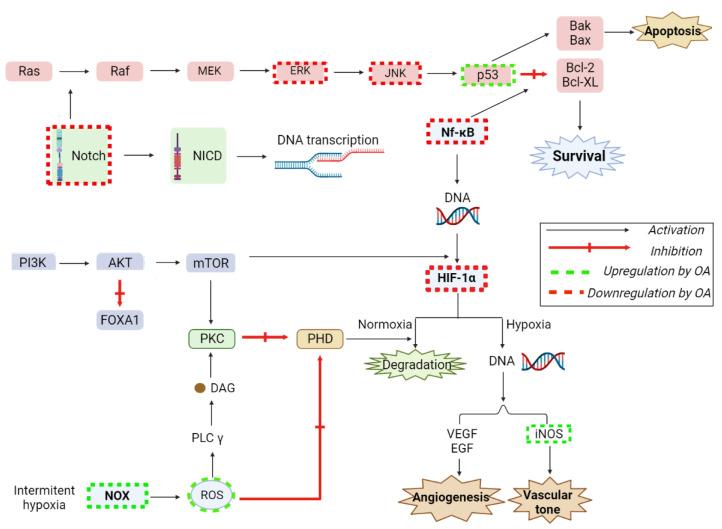
Schematic representation of the reported anticancer mechanisms of OA; key signaling pathways targeted by OA in cancer: PI3K/Akt pathway up-regulation -> inhibition of cell migration; Notch signaling pathway inhibition -> inhibition of cell proliferation; NF-kB signaling pathway suppression -> anti-inflammatory effect; ERK/JNK/p38 pathway activation -> induction of apoptosis. Created with BioRender.com (accessed on 8 May 2022).

Thyroid cancer is the most common endocrine malignancy whose certain undifferentiated forms (anaplastic carcinomas) show small survival expectancy, not exceeding 6 months. One molecular signaling pathway responsible of thyroid cancer is the overexpression of FOXA1–a transcription factor of the Forkhead box (FOX) protein family, and a pioneer factor that facilitates oncogenic transcription factors; its hyperreactive signaling due to gene amplification or overexpression was reported in many cases of thyroid lesions and tumors [123]. Duan L. et al. studied the in vitro anticancer effects of OA on SW579 human thyroid carcinoma cell lines; the molecular mechanism involved was revealed as the inhibition of the FOXA1 signaling pathway that succeeded in reducing the viability of SW579 cell lines in a dose-dependent manner. FOXA1 can be found in several organs, including thyroid tissues; its gradual overexpression was reported in advanced stages of thyroid carcinomas. OA down-regulated the expression of FOXA1 by activating the PI3K/Akt pathway (Figure 10), hence decreasing the vimentin protein expression, blocking the migration, invasion, and colony formation of SW579 cell lines [124]. 

OA also exerted anticancer effects on rectal cancer cell lines through several molecular mechanisms; it stimulated the reactive oxygen species (ROS) and NADPH oxidase 2 (NOX2) and inhibited the overexpression of HIF-1α in a dose-dependent manner, leading to G1/S cell arrest and, subsequently, to the inhibition of rectal cancer cells proliferation [125]. HIF-1α is one of the two subunits of hypoxia-inducible factor-1 (HIF-1), which after activation plays a crucial role in the adaptive response of tumor cells growth by stimulating the overexpression of glucose transporters (GLUTs) involved in meeting the energetic needs of tumor cells. Furthermore, HIF-1 promotes the overexpression of vascular endothelial growth factor (VEGF) and oncogenic growth factors such as transforming growth factor beta 3 (TGF-β3) and epidermal growth factor (EGF) [126]. It was reported that OA up-regulated NOX2 expression, which led to the inhibition of HIF-1α expression, thus triggering the inhibition of cell proliferation in several rectal cancer cell lines and inducing G1/S cycle arrest [125]. OA also exhibited anticancer activity in colon cancer by regulating the mTOR and AMPK signaling pathways. mTOR is a protein that regulates the growth and proliferation of cancer cells by sensing nutrition levels and growth factors; it is highly expressed in colon cancers. On the other hand, adenosine monophosphate-activated protein kinase (AMPk) plays an important role in maintaining cell homeostasis and activating cell autophagy. The activation of AMPk not only can induce the overexpression of caspase-3, caspase-8, and caspase-9, that would lead to cancer cell autophagy, but also regulates the mTOR, hence inducing autophagy and apoptosis in colon cancer cells. It was reported that OA induced autophagy in vitro in SW-480 and HCT-116 colon cancer cell lines in an AMPk-dependent manner, by inhibiting the mTOR signaling pathway and leading to colon cancer apoptosis [127]. Numerous research groups have also focused on the synthesis [128] and elucidation of the MOA of semisynthetic derivatives with enhanced pharmacological properties compared to OA. Recent promising results include the induction of the intrinsic apoptotic pathway in melanoma cells (B16-F10) by -O-succinyl-28-O-benzyl-OA [129], the induction of the extrinsic apoptotic pathway in colon cancer cells (HT-29) by PEGylated OA derivatives [130], and the S cycle arrest in melanoma (B16-F10) and hepatocarcinoma cells (HepG2) by diamine and PEGylated-diamine OA derivatives [131,132], suggesting that the chemical derivatization of OA could lead to more potent molecules for therapeutic use.

OA proved its efficacy against osteosarcoma by inhibiting the proliferation and viability of human osteosarcoma cell lines Saos-2 and MG63 in a dose-dependent manner. The mechanism behind this effect resides in the inhibition of the Notch signaling pathway [133], as revealed by the high levels of Notch pathway genes (Notch ligand-delta1, Notch 1 and Notch 2) in osteosarcoma cells associated with high metastatic potential; the Notch signaling pathway plays a crucial role in cell development, survival, differentiation, and proliferation (Figure 10). After activation by ligand binding, Notch proteins are translocated to the nucleus where they interact with the transcription factor CSL (RBP-Jκ) and the mastermind-like protein (MAML). By binding to RBP-Jκ, intracellular domain of Notch (NotchIC) displaces repressors complexes, by converting RBP-Jκ from a transcriptional repressor to an activator, thus leading to the initiation of Notch genes transcription. It has been reported that the overexpression of Notch receptors controls cell invasion and metastasis through the stimulation of downstream target genes, including HES1, which acts as transcriptional regulator of osteosarcoma cells metastasis [134]. The results showed that OA inhibited the expression of Notch target gene HES1 in a dose-dependent manner, showing an anti-proliferative and proapoptotic effect in Saos-2 and MG63 cells.

OA may act as a alleviator of epithelial–mesenchymal transition (EMT), which plays a significant role in the progression of interstitial fibrosis; the phytocompound dose-dependently activates the NF-E2-related factor 2 (Nrf2) which protects tissues against oxidative stress and activates klotho protein expression; in turn, klotho acts as a direct inhibitor of TGF- β1, therefore blocking its binding to the surface receptors and reducing EMT responses [135]. The EMT attenuating effect of OA was also investigated by Wang et al. in HepG2 liver cancer cell lines; the underlying molecular mechanism consists in the stimulation of the inducible nitric oxide synthase (iNOS) activation, which in turn increases NO production. In many tumors, the overexpression of iNOS promotes cancer evolution while the elevated NO levels exert cytotoxic effects by inducing DNA damage and tumor cell death. Due to this mechanism, OA exhibits a strong anti-EMT effect in HCC, additionally potentiated by the nitrification of EMT-related proteins and reduction of EMT-markers (i.e., E-cadherin, vimentin) [136].

The anticancer activity of OA in hepatocellular carcinoma cells (HCC) is also mediated through the microRNA-122 expression [137]; microRNA-122 is a liver-specific member of the miRNA gene family that plays a significant role in cell proliferation, migration, apoptosis, and invasion. The reduction of miRNA expression is a common feature in different types of cancers; more specifically the down-regulation of miR-122 occurs in hepatocellular carcinoma metastasis, presumably due to the inhibition of the EMT pathway [138]. It was reported that OA activated the miR-122 gene expression in a dose-dependent manner, thus leading to the inhibition of HCC migration and invasion. OA also exerted an anti-metastasis effect on HCC by inhibiting the expression of EMT pathway and subsequently reducing EMT markers (β-catenin, N-cadherin, and vimentin) [137]. Hosny et al. revealed the in vitro antiproliferative activity of OA in both HepG2 and EAC cell lines as well as its in vivo autophagic and apoptotic effects on 7,12-dimethylbenzanthracene (DMBA)-induced liver carcinogenesis. In terms of molecular mechanisms, OA promoted cell apoptosis by increasing the previously DMBA-inhibited caspase-3 expression; furthermore, OA-induced autophagy and apoptosis were achieved by down-regulating Bcl-2 expression, which is responsible for autophagosome formation and autophagy down-regulation. The DMBA administration also produced a significant increase in NF-kB signaling, hence increasing the transcription of NF-kB-regulated VEGF, COX-2, and TNF-α; by suppressing the NF-kB signaling pathway (Figure 10), OA decreased the production of inflammatory factors and alleviated hepatocarcinogenesis [139]. In contrast, Zhou et al. documented the OA proapoptotic activity by the up-regulation of Bcl-2 proapoptotic protein and down-regulation of the anti-apoptotic Bax protein in SMMC-7721 hepatocellular carcinoma cells, thus significantly inhibiting HCC proliferation in a dose-dependent manner. However, OA showed an important role in SMMC-7721 autophagy through the inhibition of Akt/m-TOR autophagy pathway by down-regulating Bcl-2 protein expression and enhancing Beclin-1 expression, finally resulting in self-phagocytosis. Beclin-1 is an autophagy-related marker that participates in the modulation of the PI3K pathway, also regulating autophagic activity [140]. Similar results in terms of Bcl2 and Bax regulation were reported by Peng et al., who emphasized the activity of OA as an apoptosis inductor through the activation of the caspase cascades in human Bel-7402 hepatocarcinoma cells; in addition, OA triggered G0/G1 cycle arrest through inhibiting the cyclin D1/CDK4 pathway [141]. The above-mentioned mechanisms give OA the ability to act as an inflammatory mediator in various pathologies such as hepatic ischemia reperfusion induced-injury [142].

OA was also combined with other active principles resulting in synergistic anticancer effects; the combination of OA with Hypocrellin A (HA) was investigated by Wang et al. in HepG2 HCC cells where the anticancer mechanism resides in the modulation of the Hippo/YAP signaling pathway. The Hippo signaling pathway includes a plethora of tumor suppressor genes that regulate cell proliferation and organ growth, its overexpression being modulated by the transcriptional factors of TEA family (TEAD) and by the key transcriptional cofactor YAP [143]. The authors have previously shown that YAP overexpression leads to high proliferation in HCC cells, while its inhibition would stop HepG2 growth and induce apoptosis [144]. HA down-regulated YAP and TEAD protein levels but had little effect on their mRNA expression; in addition, the presence of OA has the ability to counteract the HA resistance of HCC cells, hence the combination exhibited a synergistic effect in HCC cells by inhibiting their proliferation and migration rates [143]. Another synergistic interaction was reported between OA and sorafenib, the only first-line systemic treatment for patients with advanced HCC; the synergistic induction of HCC death was analyzed in two human HCC cell lines, Huh7 and HepG2. In terms of molecular mechanism, OA and sorafenib acted synergically as modulators of the Bcl-2 family of anti-apoptotic proteins, specifically of the myeloid cell leukemia sequence 1 protein (Mcl-1). The experimental results showed that the Mcl-1 levels have decreased after sorafenib/OA co-treatment administration, while the levels of proapoptotic proteins Bak increased. OA also led to DNA fragmentation by activating the release of cytochrome C, which enhances mitochondrial membrane disruption and finally leads to cell apoptosis [145].

Khan et al. assessed the anticancer activity of OA in hepatocarcinoma by co-loading cisplatin and OA in calcium carbonate nanoparticles (CDDP/OA-LCC NPs) and analyzing their pro-apoptotic effect in HepG2 cell lines. The authors reported that CDDP/OA-LCC NPs exerted a synergistic pro-apoptotic effect compared to the free drug solutions and blank calcium carbonate nanoparticles. The molecular mechanism responsible for the apoptotic effect consisted in the down-regulation by OA of the PI3K/AKT/mTOR pathway through the inhibition of mTOR phosphorylation triggered by the AMPK pathway activation, resulting in the inhibition of cell proliferation and survival. In addition, OA up-regulated the p53 proapoptotic pathway through the up-regulation of the pro-apoptotic proteins (Bax, cytochrome C, caspase 3) finally leading to an AMPK-dependent apoptotic effect. Furthermore, OA helped overcoming drug resistance by down-regulating the NF-kB pathway which enhances the apoptotic potential of chemotherapeutic agents such as cisplatin, thus leading to drug resistance in cancer cells [146].

Kim et al. conducted a study on the anticancer activity of OA in DU145 prostate cancer cells, MCF-7 breast cancer cells, and U87 human glioblastoma cells. In terms of molecular mechanisms, OA exerted an apoptotic and anti-proliferative effect on all cancer cells by stimulating the expression of proapoptotic proteins p53 (Figure 10), cytochrome C, Bax, and caspase-3. In MCF-7 cells OA increased the expression of pro-apoptotic proteins, p21 and p53, and decreased the expression of cell cycle proteins (i.e., cyclin D, cyclin E, CDK2, and CDK4) in a dose-dependent manner, leading to cycle arrest in various phases [147].

OA was found active against gastric cancer through the modulation of regulatory T cells and proinflammatory T helper 17 (Treg/Th7). In terms of molecular mechanism, OA exerted a regulatory effect on the Treg/Th17 imbalance by targeting IL-6 through the overexpression of miR-98-5p. Treg cells possess a negative regulatory effect on immunity by inhibiting the killing effect of T cells on tumor cells, this imbalance being observed in several malignancies. miR-98-5, belonging to the miRNA family and being involved in many regulatory processes, was reported to have a binding site to Interleukin-6 (IL-6); IL-6 is a cytokine that modulates the generation of Th17 cells from native T cells, and is also overexpressed in gastric cancer [148]. The antiproliferative activity of oleanolic and UAs extracted from *Actinidia chinensis* was tested both in vitro in HGC-27 gastric carcinoma cell lines and in vivo on a zebrafish xenograft model, exhibiting a strong apoptotic effect on cancer cells in direct proportion to the increase in phytocompounds’ concentrations. The molecular mechanism that may explain the anticancer activity is the up-regulation of cytochrome C, which in turn modulates the cleaved caspase 3, hence inhibiting HGC-27 proliferation and stimulating their apoptosis [149]. Cytochrome C is an important component of the mitochondrial membrane as an electron carrier, which after cytosol release activates the apoptotic protease activating factor-1 (Apaf-1), which forms the apoptosome, a key activator of caspases 3, 6, and 7 with major roles in cell apoptosis [150]. The combination of OA and UA was also tested for their anticancer effect on A549 lung cancer cells through autophagy; the molecular mechanism involves several pathways. The PTEN-induced kinase 1(PINK1)/Parkin signaling pathway mediates the mitophagy process; PINK1 kinase is induced by mitochondrial fragmentation and directly phosphorylates Parkin, thus triggering autophagy. The two triterpenic acids induced autophagy in vitro on A549 cells independently of Parkin activation; moreover, UA proved to down-regulate the AKT/mTOR pathway by mediating the PI3K/AKT pathway. In comparison, OA triggered autophagy through inhibiting the p-mTOR expression, but independently of AKT; it was also noticed that the production of reactive oxygen species (ROS) induced by treatment with OA and UA, facilitated the autophagy process, acting as a trigger in the induction of PINK1/Parkin pathway by altering mitochondrial membrane potential [151].

The anticancer activity of OA combined with esculetin against cervical cancer was tested in vitro by Edthara et al.; OA has been reported to inhibit the major signaling pathways like PI3K, Akt, ERK/JNK/p38, mTOR, NF-kB, and S6K, while esculetin was reported to induce cell death by activating various pathways such as JNK, ERK, Caspase-3, and NF-kB (Figure 10). The combination of the two active compounds tested in vitro on HeLa cells exhibited synergic apoptotic effects by increasing the levels of pro-apoptotic and tumor suppressor genes BAX and PTEN and inhibiting the level of STAT-3 [152]. STAT-3 belongs to the family of signal transducer and activator transcription (STAT) proteins, its high levels being prevalent in different types of cancer. STAT-3 may be activated by tyrosine phosphorylation in response to several cytokines signals, contributing to tumor proliferation and survival, thus representing a promising direct target for novel anticancer therapies [153].

### 3.2. Antidiabetic Activity

**Figure 11:**
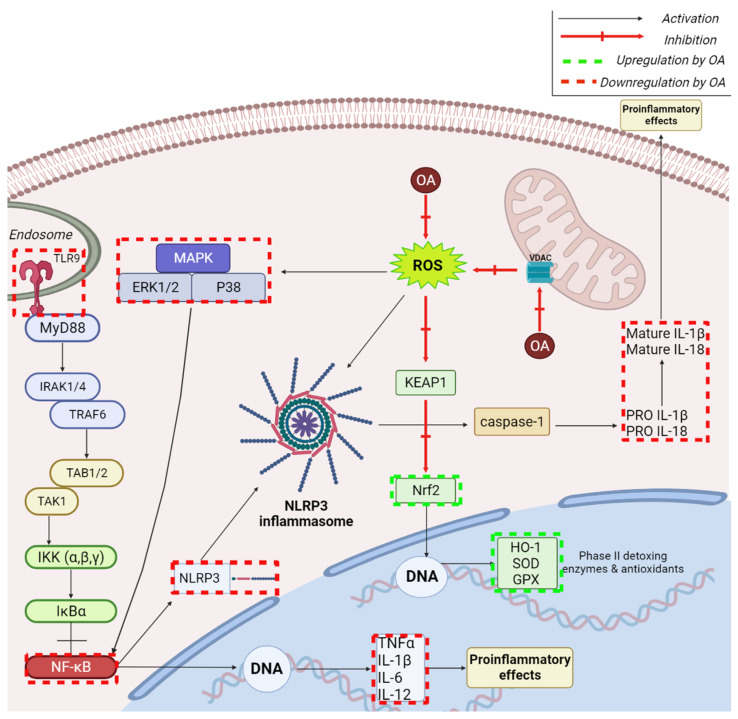
Schematic representation of the reported antidiabetic mechanisms of OA; key signaling pathways targeted by OA in diabetes: NF-kB signaling inhibition -> reduces IL-6 and TNF-α inflammatory cytokines -> antioxidant effect; NLRP3 inflammasome inhibition -> reduces expression of pro-inflammatory cytokines -> anti-inflammatory effect; MAPK signaling inhibition -> antioxidant effect; HO-1/Nrf2 pathway up-regulation- antioxidant effect. Created with BioRender.com (accessed on 8 May 2022).

The overexpression of inflammation markers is correlated to diabetes-associated complications, including the progression of vascular diseases; An et al. evaluated the effect of OA in alleviating carotid artery injury in streptozotocin-induced diabetic rats. In terms of molecular mechanism, OA exhibited a strong anti-inflammatory activity by inhibiting the expression of NF-kB signaling as well as of IL-6 and TNF-α inflammatory cytokines; moreover, OA increased the production of antioxidants by stimulating the Nrf2 signaling pathway (Figure 11). OA exhibited an alleviating effect on rats carotid artery injury by inhibiting the mRNA and protein expression of NLRP3 inflammasome, leading to the suppression of caspase-1 and IL-1β [154]. NLRP3 (NOD-, LRR-, and pyrin domain-containing protein 3) is an endogenous sensor that detects a broad spectrum of environmental irritants, leading to the activation of the NLRP3 inflammasome multiprotein complex. Once activated, the NLRP3 inflammasome stimulates the formation of cysteine protease caspase-1 that releases the pro-inflammatory cytokines IL-1β and IL-18, which will be subsequently cleaved by caspase-9 and turned into their active forms (Figure 11) [79]. The inhibitory activity of OA on pro-inflammatory cytokines may ameliorate the progression of pre-diabetes state into type 2 diabetes by diminishing subclinical inflammation. OA significantly reduces the expression of TNF-α, IL-1β, NF-kB, and CD40L in pre-diabetic animals; CD40L is an inflammatory mediator belonging to the TNF family that interacts with immune cells such as T cells and induces the production of chemokines, leading to inflammatory responses [155]. A frequent complication of diabetes is the increased incidence of chronic kidney disease; Gamede et al. have continued their research by assessing the alleviating effect of OA on kidney-affected pre-diabetic rats. The results showed that OA reduced oxidative stress within the kidney, deactivated the renin-angiotensin-aldosterone system (RAAS), and normalized the albumin/creatinine ratio as well as the glomerular filtration rate. The ROS are frequently associated with the damage of the podocyte layer of the glomerular membrane, which prevents nephron protein filtration, leading to proteinuria; OA up-regulated superoxide dismutase (SOD) and glutathione peroxidase (GPx), leading to a significant decrease in lipid peroxidation, urine podocin, and albumin/creatinine ratio, thus preventing kidney injuries and proteinuria. Moreover, OA was able to reduce mean arterial pressure by deactivating the RAAS system and suppressing sodium reabsorption [156].

The protective activity of OA against induced diabetes in rats was also approached by Iskender et al.; OA was able to ameliorate the effects of diabetes by inhibiting the pro-inflammatory cytokines toll-like receptor-9 (TLR-9), IL-18, NF-kB, and malondialdehyde (MDA) levels. TRL-9 is a member of the TLR family that is involved in the immune response by activating the production of proinflammatory cytokines; it possesses an important role in regulating the development and function of pancreatic β-cells. Once activated, TRL signal pathways activate NF-kB, leading to the overexpression of pro-inflammatory cytokines (Figure 11). Serum MDA concentration reveals the level of lipid oxidation produced by hyperglycemia due to the destruction of pancreatic β-cells. Therefore, OA was able to reverse the adverse effects induced by diabetes, ameliorating both lipid and mineral serum concentration and normalizing blood sugar levels [157].

Obesity is a risk factor for diabetes due to its association with chronic inflammations; Li et al. revealed that the anti-inflammatory effect of OA on obesity-induced inflammation in mice is presumably caused by the suppression of interferon gamma/lipopolysaccharide (IFN-γ/LPS)-induced M1 phosphorylated kinases by reducing ROS expression, hence inhibiting the MAPK signaling and NLRP3 inflammasome formation (Figure 11) [158]. Another diabetes-associated pathology is cardiomyopathy, which may occur due to an inefficient treatment management; the phytocompound is able to improve the myocardial cell damage while fighting oxidative stress. The underlying molecular mechanism consists in the up-regulation of the HO-1/Nrf2 pathway (Figure 11) accompanied by the modulation of the insulin phosphorylated-glycogen synthase (GS)/glycogen phosphorylase (GP) pathway. Experimental results showed that OA exhibited a protective effect against cardiomyopathy by inhibiting the oxidative stress through promoting the HO-1/Nrf2 pathway and simultaneously reduced blood glycogen levels by modulating the GS/GP pathway [159].

Polychlorinated biphenyls exposure is frequently associated with obesity and diabetes; Su et al. have emphasized the ability of OA to inhibit oxidative stress induced in Aroclor 1254-treated mice. The authors reported that OA decreased ROS, NOX expression, and PPARγ signaling and increased hepatocyte nuclear factor 1B (HNF1b) expression in mouse adipocytes. HNF1b plays a key role in the hepatic fat synthesis regulation, glucose homeostasis, and insulin resistance; it also activates the antioxidant enzyme transcription and suppresses the transcription of oxidant enzymes such as NADPH oxidase 1 (NOX) while down-regulating the expression of PPARγ in the process of adipocyte differentiation [160]. PPARγ belongs to the wide family of PPARs (peroxisome proliferator-activated receptors), which, after being activated through binding with a responsive regulatory element, control the expression of the genes involved in adipogenesis, lipid metabolism, and inflammation. PPARγ is highly expressed in white adipose tissue and possesses a key role in adipogenesis by modulating the body lipid metabolism and insulin sensitivity [161]. 

It is well known that poor diabetes management with frequent episodes of high blood glucose levels leads to impaired endothelial functions resulting in insufficient NO production; Zhang et al. have analyzed the ameliorative action of OA against endothelial dysfunction. OA activated the PPARγ target genes (PDK4, ADPR, and ANGPTL4) and modulated the eNOS/Akt/NO pathway, which is involved in NO production through the oxidation of L-arginine; the eNOS activity is modulated by Akt that induces eNOS-Ser phosphorylation leading to NO production in response to diverse stimuli. The NO production is also stimulated by PPARγ and leads to endothelial vasodilation. OA was able to restore the endothelial function impaired by high glucose levels due to the activation of the PPARγ-mediated Akt/eNOS signaling pathway [162].

An important aspect of diabetic homeostasis is the maintenance of glycemic control through the inhibition of α-glucosidase activity; α-glucosidase is a carbohydrate hydrolase that regulates blood sugar by hydrolyzing the 1,4-α-glucopyranosidic bond, leading to α-glucose production, hence increasing the blood sugar levels [163]. Molecular docking studies showed that the combination of oleanolic and UAs inhibited the activity of α-glucosidase by binding preferentially to different allosteric sites, interacting with surrounding amino acid residues, and disturbing the enzyme conformation, finally resulting in a decrease in α-glucosidase activity [164]. 

The combination of OA and UA was able to treat disorders in carbohydrate uptake through inactivating human salivary α-amylase (HSA), an important enzyme in starch and glycogen digestion. The two compounds have shown non-competitive inhibition against HSA, but the inhibitory ability of OA is lower than that of UA. The crucial residues, Arg 195 and Asp 197, might explain the enzyme interaction with all inhibitors by hydrogen bonds; UA was able to form two hydrogen bonds with Arg 195 because of the adjacent methyl groups of the E ring, thus forming additional contacts involving the hydrophobic portion of Asp [165].

### 3.3. Antiinfectious Activity

OA exhibits strong antiviral activity against the Herpes simplex virus (HVS), both acyclovir (ACV)-sensitive and ACV-resistant HSV-1 strains. In terms of molecular mechanism, OA demonstrated antiviral activity in the early stages of the HSV infection by suppressing UL8, the critical helicase-primase complex for viral replication. UL8 is a subunit of the helicase-primase heterotrimer that interacts with the other two components, UL5 and UL25, and modulates the formation of the UL5/UL25 subcomplex, leading to viral replication [166].

Combining antibiotics with anti-resistance compounds has become a new strategy for fighting antibiotic resistance; Catteau et al. have analyzed the synergistic effect of OA and UA, respectively, with beta-lactams against methicillin-resistant Staphylococcus aureus (MRSA). Both UA and OA exhibited antibacterial effects in a synergistic manner with β-lactams (ampicillin and oxacillin) and inhibited the β-lactamase activity of MRSA by delocalizing PBP2 from the septal division site and inhibiting peptidoglycan synthesis [167]. Penicillin-binding protein 2a (PBPA2) is a unique transpeptidase that is not inhibited by β-lactam antibiotics and continues to catalyze cell-wall crosslinking, hence being responsible of bacterial resistance [168]. Moreover, in a murine in vivo experiment of subcutaneous MRSA-induced infection, the co-administration of UA with nafcillin reduced lesion size by inhibiting the expression of the IL-1β pro-inflammatory cytokine [167]. Another experiment reported that both OA and UA exhibited antibacterial properties by altering the fluidity and permeability of the MRSA membrane in a dose-dependent manner; moreover, UA was able to delocalize PBP2 by the disintegration of the cardiolipin-enriched domains, leading to the inhibition of the peptidoglycan synthesis [169].

### 3.4. Lipidemic Activity

Lin et al. have studied the lipolytic effect of OA through exploring its ability to inhibit Liver X receptor α (LXRα) in vitro in HepG2 cell lines. Liver X receptor α (LXRα) belongs to the activated nuclear receptor (NR) family and is highly expressed in organs responsible for cholesterol homeostasis; it activates the hepatic lipogenic pathway by stimulating the transcriptional region of LXR response element, leading to the up-regulation of target genes and subsequently up-regulating lipogenesis. OA exhibited an inhibitory effect on the LXR response element by diminishing its transactivation as well as on sterol regulatory binding protein-1c (SREBP-1c); moreover, OA was able to reduce mRNA and protein expression of LXRα target genes, subsequently decreasing lipid accumulation in hepatic cells. Furthermore, OA was able to down-regulate valproate and rifampicin-induced LXRα, showing potential for future drug-induced hepatic steatosis treatment [170]. OA showed an alleviating effect on atherosclerosis progression in three animal models; the study revealed that OA was able to up-regulate the mRNA expression of AdipoR1, AdipoR2, and PPARγ, leading to a decrease in serum lipid levels and lipid accumulation in the liver [171]. AdipoR1 and AdipoR2 belong to the progestin and adipoQ receptor family, acting as receptors for adiponectin; AdipoR1 activates AMPK pathways, while AdipoR2 activates the peroxisome proliferator-activated-receptor-α (PPARα), both modulating glucose and lipid metabolism and oxidative stress [172]. Luo et al. have further analyzed the OA lipid-lowering effect in hyperlipidemic patients; the results showed that OA was able to modulate the expression of genes involved in lipid metabolism, thus improving hyperlipidemia, and can also modulate the expression of pro-inflammatory cytokine TNF-α, leading to the improvement of insulin sensitivity [173].

### 3.5. Cardioprotective Activity

OA exhibits a strong antihypertensive activity through phospholipid levels modulation; Zhang et al. have investigated OA properties in vivo on hypertensive rats. The lipid metabolism was regulated by OA through the inhibition of secretory phospholipase A2 (sPLA2) and fatty acid synthase (FAS), leading to improved blood pressure and lipidic profile. sPLA2 promotes foam cell formation by increasing the proinflammatory bioactive lipid levels and decreasing HDL levels; as a result, OA was able to inhibit the accumulation of triglycerides and cholesterol [174]. The antihypertensive activity can be modulated through the renin-angiotensin system and natriuretic hormone; in terms of molecular mechanism, OA was able to fight hypertension by down-regulating the renin-angiotensin-aldosterone system in the kidneys, thus inhibiting the plasmatic levels of renin, angiotensin, and aldosterone as well as up-regulating the expression of natriuretic peptide receptor C in the heart, hence improving blood pressure balance [175]. In contrast, Pan et al. reported that the anti-atherosclerotic activity of OA is induced through the up-regulation of angiotensin, which exerts a vascular protective activity by restoring endothelial function and modulating lipid metabolism. The authors also reported that OA inhibited the expression of TNF- α, IL-6, and IL-1β pro-inflammatory cytokines, leading to the up-regulation of angiotensin, NO, and eNOS both in vitro and in vivo [176].

### 3.6. Renal Activity

Benign prostatic hyperplasia (BHA) is one of the most frequent diseases in men over 50; OA was found to exert efficient ameliorating effects in vitro in BPH-1 cell lines and in vivo against a testosterone-induced BHA animal model. OA showed suppressive effect on testosterone-induced BPH by inhibiting in vitro cell cycle progression through the down-regulation of cyclin-Cdk complexes; in vivo, OA down-regulated 5α-reductase expression, leading to a reduced DHT production and subsequently stopping prostate weight gain. Furthermore, the therapeutic effect exhibited by OA was similar to the one obtained following finasteride treatment, proving OA as a future candidate for BHA treatment [177]. Yang et al. have investigated OA action against renal ischemia reperfusion injury (RIRI) in vivo; the molecular mechanism resides in OA’s ability to down-regulate the PI3K/AKT signaling pathway, hence inhibiting the release of ROS and ameliorating the effects of RIRI [178]. Renal fibrosis that may occur following ischemic injuries may be alleviated by OA as a result of TGF-β/Smad pathway inhibition induced by down-regulating the expression of TGF-β receptor I and TGF-β receptor II and by inhibiting Smad phosphorylation [179].

Acting as an antiinflammatory agent, OA was found to prevent aging-related testicular dysfunction by inhibiting several pro-inflammatory cytokines, such as IL-1β, COX-2, and TNF-α, and leading to an improved testicular weight and index. Further in-depth studies revealed that OA inhibited the phosphorylation of NF-kB and attenuated DNA damage and apoptosis through the modulation of the Bcl-2/Bax ratio as a result of Bcl-2 up-regulation combined with Bax down-regulation [180].

### 3.7. Anti-Osteoporotic Activity

OA proved useful in bone tissue regeneration by modulating Notch signaling and growth factor bone morphogenetic protein 2 (BMP2); the phytocompound induced mesenchymal stromal cell (MSC) osteogenic differentiation in a dose-dependent leading to the enhanced osteogenic proteins expressions of ALP, Runx2, and Collagen I, and subsequently stimulating bone formation [181]. Additionally, in vitro and in vivo studies showed that OA inhibited TNF-α, MAPK, and PI3-Akt signaling pathways, the results of which were further validated through molecular docking, leading to increased bone salt deposition and accelerated calcification, and hence to an improved bone architecture [182]. In contrast, having investigated OA bone protective effects in vivo in lipopolysaccharides-induced bone loss in mice, Zhao et al. revealed that OA inhibited the receptor activator of nuclear factor kappa-B ligand (RANKL) levels, but without influencing the RANKL-induced up-regulation of the NF-kB/Akt pathways. The results indicated that OA significantly suppressed RANKL-activated osteoclast genes, leading to bone protective effects by modulating both osteoclastogenesis and osteoblastogenesis [183]. The same authors have further analyzed the anti-osteoporotic effects of OA both in vitro and in vivo, showing that OA was able to decrease osteoclast densities through RANKL modulation, and also down-regulated the osteoclast genes Tartrate resistant acid phosphatase (TRAP), Cathepsink (Ctsk), and Matrix metalloproteinase 9 (MMP9) levels, leading to osteoclastogenesis inhibition [184].

An efficient OA effect against osteoclastogenesis and bone resorption can be explained by its ability to up-regulate the expression of estrogen receptor-alpha (Erα) by direct binding, causing the up-regulation of miR-503; subsequently, miR-503 inactivates the mRNA of RANK, hence inhibiting osteoclastogenesis. ERα/β modulates gene transcription by binding to estrogen response element (ERE) and is an essential site for estrogen to express its anti-osteoporotic effect [185].

### 3.8. Antiinflammatory Activity

Osteoarthritis is a common joint degenerative disease, manifested in cartilage degradation, inhibited bone remodeling, and synovial inflammation. The molecular mechanism responsible for osteoarthritis degeneration is the stimulation of inflammatory cytokines and active metalloproteinases (MMPs) by fibroblast-like synoviocytes (FLS) production of chemokines and cytokines. Bao et al. have reported that OA may stop osteoarthritis evolution by inhibiting synovial inflammation through the up-regulation of sirtuin 3 (SIRT3) expression, leading to the inactivation of the NF-kB pathway [186]. SIRT3 is a major deacetylase in mitochondria; it prevents apoptosis by down-regulating ROS expression and preventing ROS-induced COX2 overexpression and oxidative stress activation, hence limiting the imbalance between anabolism and catabolism in FLS [187]. The osteoarthritis treatment may also benefit of the OA’s inhibitory activity on IL-1β-induced chondrocyte dysfunction through the down-regulation of caspase 3 production, which induces apoptosis inhibition; in addition, by up-regulating miR-148-3p gene expression OA is able to alleviate cell membrane damage [188].

In terms of molecular mechanism, the inflammatory reaction is mediated by pro-inflammatory cytokines, which in turn are modulated through the NF-kB and STAT1 signaling pathways; they play a major role in the immediate response in anaphylactic shock and lead to direct effects on tissues and mucus production. OA exhibited an anti-allergic activity by inhibiting the formation of pro-inflammatory cytokines through blocking the phosphorylation of STAT1 and suppressing p65 NF-kB translocation, thus causing the inhibition of allergic inflammatory response [189]. Furthermore, the authors have explored the anti-inflammatory effect of OA in 1-chloro-2,4-dinitrochlorobenzene (DNCB)-induced dermatitis in animal models. The phytocompound effectively inhibited the TH2 type cytokines and chemokines which up-regulate IL-4, IL-5, IL-9, and IL-13 inflammation mediators involved in humoral immunity and allergic responses [190] and down-regulated the TNF-α/IFN-γ pathways by blocking the phosphorylation and activation of Akt, NFkB, and STAT1, hence proving to be a suitable candidate for allergic disorders [191]. 

An interesting approach of the anti-inflammatory effect of OA was conducted by Dong et al. in Salmonella typhimurium-induced diarrhea in mice; the authors reported that OA was able to alleviate the intestinal inflammation and barrier damage by reducing the levels of COX-2 and iNOS, and also down-regulated the IL-1β, IL-6, and TNF-α pro-inflammatory cytokines levels; the inhibition of pro-inflammatory cytokines also prevents the acetaminophen- or concanavalin A-induced necrosis of hepatic tissues [192,193]. Furthermore, the experimental data showed that OA significantly reduced IκB phosphorylation, leading to the down-regulation of NF-κB and MAPK signaling pathways and subsequently reducing the intestinal inflammation [194]. The same molecular pathways have been explored by Phull et al. in order to establish OA effects on inflammation and cellular differentiation of chondrocytes; interestingly, the results showed that OA exhibited a significant anti-inflammatory activity by increasing COX-2 expression through the inhibition of ERK and decreasing the p38 cascade, leading to the revival of type-II collagen [195]. In contrast, Peng et al. showed an inhibitory activity of OA on collagen formation in silica-induced lung injury and fibrosis; an in-depth research showed that OA inhibited the phosphorylation of AKT1, thus inducing the inactivation of NF-kB and down-regulation of pulmonary cytokines levels, subsequently stopping silicosis progression [196]. In addition, OA significantly stimulated the anti-oxidant transcription factors SIRT1, Nrf2, and Bcl-2 anti-apoptotic protein expression and down-regulated the expression of NF-kB inflammatory signaling pathway and Bax protein, leading to reduced lung epithelial cell apoptosis and ameliorated lung injuries [197]. Similar effects were reported against spinal cord injury where the inhibition of the IKK/NF-kB pathway caused the suppression of apoptosis and inflammation in murine models [198].

### 3.9. Neuroprotective Activity

OA exerts a profound protective effect against early brain injury after subarachnoid hemorrhage where the extracellular HMGB1 can induce cytokine release and leukocyte recruitment to trigger inflammatory response. Oleanoic acid can reduce the level of serum HMGB1 by inhibiting the transfer of HMGB1 from nucleus to cytoplasm, thus reducing early brain injury and neuronal apoptosis after subarachnoid hemorrhage. On the other hand, SIRT1, one of the most studied deacetylases, inhibits the translocation of HMGB1 to the cytoplasm by deacetylating HMGB1 lysine, thereby reducing its serum level; OA acts as SIRT activator, thus enhancing HMGB1 deacetylation and then inhibiting the transfer of HMGB1 from nucleus to cytoplasm, thereby inhibiting inflammation [199].

In addition to anti-inflammatory properties, OA exerts a potent antioxidant activity and protects cells and tissues against oxidative stress; the protective effects of OA against oxidative stress in Alzheimer’s disease were mediated via regulation of STC-1 and UCP2 signaling pathways. UCP2 inhibition significantly reversed the regulatory effect of OA on cell viability, caspase-3 activity, reactive oxygen species content, and β amyloid level in N2a/APP695swe cells [200]. An essential process in the pathological events occurring in individuals with Alzheimer’s disease was also linked to abnormal amyloid β accumulation and deposition in the hippocampus. It should be emphasized that OA exhibited a positive effect on preventing neuron injury, maintaining synaptic integrity, and improving the neuron arrangements. Furthermore, OA could increase the N-methyl-D-aspartate receptor, calmodulin-dependent-protein kinase II, and protein kinase C protein expression in order to restore and maintain synaptic plasticity. In addition, the authors revealed that OA could enhance the synaptic transmission by modulating the expression of brain-derived neurotrophic factor, tyrosine kinase B, and cAMP response element-binding protein [201].

OA may also act as a neuroprotective agent against neurodegenerative pathologies such as Parkinson’s disease by attenuating striatal microglial activation. The ability of OA to attenuate 6-hydroxydopamine-induced intracellular reactive oxygen species and prevent cell death in PC12 cells is not clearly understood. Some key data suggest that OA may interact directly with microglia, thus attenuating microglial activation that would result in an unremitting neuroinflammatory response. Furthermore, this phytocompound removes intracellular ROS, preventing microglial migration toward neurons; nonetheless, the study demonstrated the role of alfa-Synuclein in microglial migration regulation via hydrogen peroxide, showing that it binds to CD11b, a receptor expressed in microglia, and leads to subsequent neuronal damage [202]. The antioxidant effect of OA, indicated by lowered generation of ROS and MDA, may provide a short-term protection against middle cerebral occlusion induced ischemia-reperfusion injury; OA facilitated the activation of Nrf2 signaling and up-regulation of its downstream stress response protein HO-1 in the injured cortex. Activation of Nrf2 protects neurons from microglia-induced oxidative damage by repressing the expression of neuronal nitric oxide synthase while the up-regulated HO-1 protect neurons in the early stage of ischemia as well as astrocytes and microglia in the later stage of ischemia from oxidative stress. The long-term protective effect is attributed to its roles in neuroglia modulation, promoting synaptic connections and promoting nerve regeneration [203]. After treatment with OA, neural stem cell (NSC) induction was mediated through GSK3β, an important target in the regulation of NSC proliferation and differentiation. Inhibition of GSK3β activity by phosphorylation at Ser9 accelerated the nuclear translocation of unphosphorylated active β-catenin, significantly promoting NSC proliferation and neural differentiation. In addition, OA could dose-dependently induce the differentiation of NSCs into neurons, while suppressing the formation of astrocytes [204].

The orofacial antinociceptive effect of OA was revealed through the possible modulation of TRPV1 receptors; TRPV1, a nonselective cation channel, may be activated by a wide variety of stimuli. The antinociceptive effect of OA was significantly reduced in adult zebrafish pre-treated with ruthenium red, a TRPV1 receptor antagonist; furthermore, when a TRPV1 channel antagonist is used, the antinociceptive response is blocked. In addition, depletion of TRPV1 + C fibers by capsaicin eliminated the analgesia produced by OA; collectively, the inhibition of TRPV1 channel by OA confirms its potential pharmacological relevance against acute pain [205].

### 3.10. Other Biological Activities

Hepatoprotection is one of the most notable pharmacological effects of OA. Low doses of OA protect against hepatotoxicity induced by a lot of stimuli, such as carbon tetrachloride, acetaminophen, cadmium, and phalloidin. The marked activation of Nrf2 by OA could explain, at least in part, the OA hepatoprotection against the above-mentioned toxic stimuli. In addition to Nrf2 activation, other mechanisms, such as a decrease in CYP2E1, decrease in Oatp1b2, modulation of the immune response, and inflammation may offer hepatoprotection. Furthermore, OA may act as a TGR5 agonist, but TGR5 activation is also associated with risks of histopatological changes, tumorigenesis, and pruritus. Higher doses can produce liver injury, including cholestasis, but this paradoxical effect could also depend on coexistent pathological conditions [206].

Further studies have shown that OA would suppress the cellular production of resistin in adipocytes by specifically blocking the Tyk2-STAT1 pathways in the middle phase of adipocyte differentiation. The completion of adipocyte differentiation was altered by the phytocompound by suppressing SOCS3, whose cellular expression is up-regulated during the later phase of differentiation; OA also suppressed PPARγ induction, revealing that the JAK-STAT signaling pathway was not activated during the completion of adipocyte differentiation [207].

OA proved useful in androgenic alopecia in vitro models by inhibiting the activity of 5α-reductase activity and regulating the alopecia-correlated genes expressions. Testosterone is converted into dihydrotestosterone (DHT) by 5α-reductase, being responsible for androgenic alopecia, by increasing TGF-β1 and TGF-β2, leading to a down-regulated proliferation of epithelial cells, and by up-regulating Dickkopf related protein-1 (DKK01) causing an inhibitory effect on epithelial cells growth in hair follicles. The results showed that OA inhibited the activity of 5α-reductase in vitro in a dose-dependent manner, and also reduced DHT production, leading to dermal papilla cells proliferation and reduced impact of DHT on gene expression [208].

## 4. Ursolic Acid

**Figure 12:**
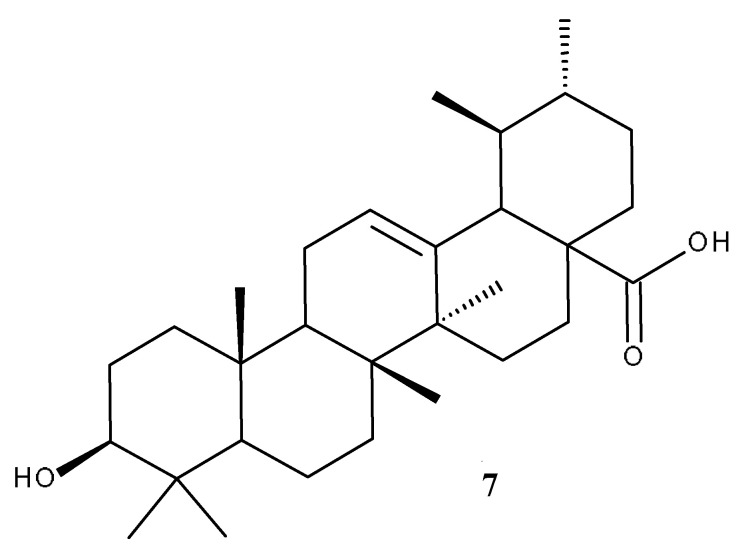
The chemical structure of ursolic acid (**7**).

Ursolic acid (UA, Figure 12) is abundantly found in nature in numerous fruits and vegetables, dietary fibers, and in the herbs of the *Lamiaceae* family, possessing little to no toxicity [209]. UA exerts a plethora of pharmacological activities such as anti-cancer, anti-inflammatory, anti-diabetic, neuroprotective, antimicrobial, and anti-hypertensive properties [210].

**Figure 13:**
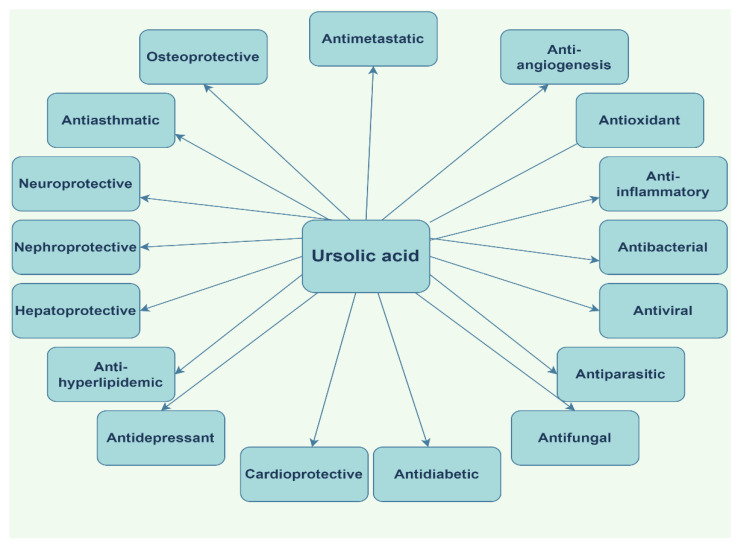
Biological activities of ursolic acid.

One excellent review on UA was published in 2015 by Kashyap et al., comprehensively describing its biosynthesis, pharmacokinetics, and pharmacodynamics properties as well as its molecular mechanisms. UA was revealed as an effective biologic agent with various therapeutic properties (Figure 13) that have become useful in many conditions [211]. 

### 4.1. Anticancer Activity

**Figure 14:**
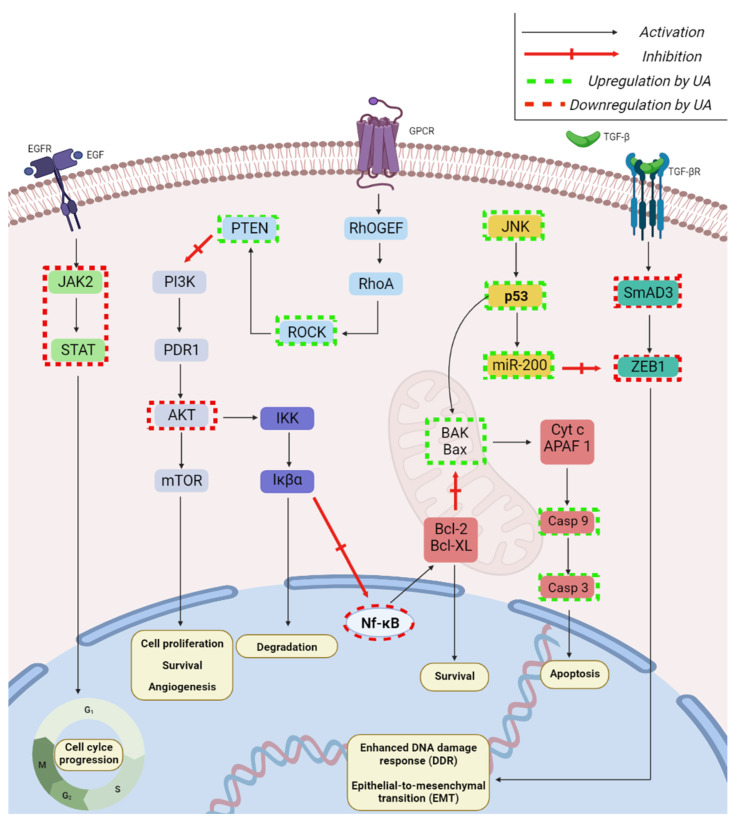
Schematic representation of the reported anticancer mechanisms of UA; key signaling pathways targeted by UA in cancer: PI3K/Akt signaling pathway inhibition -> inhibition of angiogenesis, cell survival, and proliferation; JNK signaling pathway activation-> induction of apoptosis; TGF-β1 pathway inhibition -> miR200a/b/c up-regulation -> induction of apoptosis; EGFR/JAFK2/STAT3 signaling pathway down-regulation -> G0/G1 cycle arrest -> apoptosis; ROCK1/PTEN/cytochrome c signaling pathway down-regulation-> activation of caspases -> induction of apoptosis; IKK/NF-kB pathway down-regulation -> G0/G1 cycle arrest -> apoptosis. Created with BioRender.com (accessed on 8 May 2022).

UA acted as an anticancer agent in vitro and in vivo in C6 glioma cells and glioma murine models, respectively, by inhibiting cell proliferation and inducing apoptosis. It was reported that UA induced apoptosis in a dose-dependent manner in C6 glioma cells by increasing the expression of sub-G1 fractions (validated as marker for apoptotic cells); the apoptotic cell death was achieved through the conversion of the inactive pro-enzyme into active caspase 3, thus initiating caspase cascade. Furthermore, UA inhibited the activation of Akt by decreasing its phosphorylation, hence stopping cell survival and proliferation (Figure 14). In vivo, UA treatment proved to decrease tumor growth; however, in all tested concentrations, the phytocompound did not decrease the malignant feature but did not alter the animal biological parameters either, hence proving its safety for future research using higher doses [212]. Similarly, UA induced significant cytotoxic effects in U-251 MG multiforme glioblastoma cells where the apoptotic cell death was dose-dependent following the regulation of the JNK signaling pathway (Figure 14), which led to mitochondrial depolarisation and lysosome accumulation; low UA doses inhibited the proliferation of U-251 cell lines, while higher doses exhibited pro-apoptotic effects [213]. Jun N-terminal kinases or JNKs belong to the MAP-kinases family and are involved in the regulation of mitochondrial apoptotic pathways by activating pro-apoptotic genes and modulating the activities of pro- and anti-apoptotic proteins through phosphorylation [214]. 

The apoptotic and antiproliferative activities of UA against colorectal cancer was examined by Cai et al. in vitro in HT-29, HCT-8, and SW620 human colon carcinoma. In terms of molecular mechanisms, the authors analyzed the protein sequencing and signal transduction modulations effects on cellular proliferation, apoptosis, and cell cycle and revealed the ATP5B/CALR/HSP90BI/HSBI/HSPDI signaling network as a potential target for the phytocompound [215]. The authors have previously demonstrated that the apoptotic effect of UA on colon carcinoma cells occurs through the modulation of various signaling pathways such as STAT3, ERK, JNK, and p38MAPK [216]; the modulation of STAT3 and miR-4500 by UA was also reported by Kim et al., who established that UA inhibited the phosphorylation of STAT3 by JAK2, hence suppressing cell proliferation and up-regulating MiR-4500, which acts as tumor suppressor [217]. Furthermore, UA inhibited the proteins involved in nucleotide mechanism (ATP5B, TUBB2C), gene expression (CALR, NPM1), and apoptosis (cytokeratin 19, HSPB1, HSPA9, HSPD1) thus inhibiting cell proliferation and inducing apoptosis [215]. Another UA apoptotic molecular mechanism against colorectal cancer was reported by Zhang et al.; UA exhibited in vitro anticancer properties in HCT116 and HCT-8 cell lines by mediating the transforming growth factor- β (TGF-β1)/zinc finger E-box-binding homebox (ZEB1) pathway and the microRNA (miR)-200a/b/c (Figure 14). UA down-regulated the expression of key mediators in the TGF-β1 pathway including TGF-β1, p-Smad2/3, and ZEB1, leading to a decrease in N-cadherin protein expression; it significantly up-regulated miR200a/b/c and subsequently induced apoptosis in colon cancer cell lines [218]. ZEB1 is a transcriptional factor in the epithelial-to-mesenchymal transition (EMT), being in a reciprocal feedback loop with miR-200a/b/c, and influencing cellular plasticity; the overexpression of ZEB1 or TGF-β1 stimulates EMT in cancer cells, leading to tumor progression [218]. 

UA exhibited a strong apoptotic activity against non-small cell lung cancer; it dow-regulated the expression of the JAK2/STAT3 pathway and reduced the expression of VEGF and PDL-1, hence hampering the STAT3/PD-L1 complex formation and the EGFR/JAFK2/STAT3 signaling pathways. The reduction of the EGFR phosphorylation led to G0/G1 cycle arrest and apoptosis, finally resulting in the inhibition of tumor angiogenesis, migration, invasion, and metastasis (Figure 14) [219].

The previously mentioned up-regulation of the pro-apoptotic caspase proteins (caspase 3, caspase 8, and caspase 9) as well as the down-regulation of the NF-kB/Akt signaling pathway supports the anticancer activity of UA against gallbladder carcinoma where the in vitro UA anti-proliferative and proapoptotic effects manifest in a dose-dependent manner in GBC-SD cells. UA inhibition of Akt further contributed to suppressing NF-kB p-65 and mRNA levels of matrix metalloproteinase 2, hence stopping cell invasion [220].

Mu et al. demonstrated the apoptotic effect of UA in vitro on LNCaP human prostate cancer cells; UA modulated the Rho-associated protein kinase 1 (ROCK1)/phosphate and tension homolog (PTEN)- cofilin-1/cytochrome c signaling pathway (Figure 14), leading to the activation of caspase cascade and inducing apoptosis in LNCaP cells. ROCK regulates protein phosphorylation through actomyosin contraction, having an important role in extra-cellular transportation. PTEN modulates post-transcriptional regulation of encoding DNA and protein interactions, exhibiting a strong tumor inhibiting activity. Cofilin-1 regulates tumor cells metastasis; in particular, the overexpression of cofilin-1 increases the speed of tumor migration. UA targeted the ROCK/PTEN pathway that mediates mitochondrial translocation of cofilin-1; furthermore, UA stimulated the activation of cytochrome C which, combined with protease activating factor 1 (APaf-1), enhanced the formation of apoptosome and led to the activation of caspase 9 and caspase 3, subsequently resulting in LNCap apoptosis [221].

Several studies have been conducted on the potential use of UA against breast cancer, resulting in the identification of certain molecular targets. The apoptotic effects of UA were compared to the anticancer effects of betulinic acid on MCF-7, MDA-mB-231, and SK-BR-3 receptors where UA exhibited a significantly stronger activity. The molecular mechanism identified in apoptosis and autophagy resides in inhibiting the activation of the AKT signaling pathway, suppressing the phosphorylation of ERK1/2 signals and overall leading to the depolarization of mitochondrial membrane potential. Furthermore, UA may induce premature senescence in breast cancer cells by activating p21, a protein responsible for cell cycle progression at G1, hence inducing G0/G1 cycle arrest [222]. The same MCF-7 breast cancer cell line was used to reveal the UA modulating activity on the PLK1, IKK/NF-kB, and BRAF/ERK pathways; UA induced MCF-7 cells apoptosis by suppressing the phosphorylation of BRAF, ERK1, and PLK1 levels. Moreover, UA down-regulated the IKK/NF-kB pathway through inhibiting the phosphorylation activation of IKKβ, the results of which were further supported by docking analysis that suggested a binding between UA and IKKβ function domain (Figure 14) [223]. Polo-like kinase 1 (PIk1) is a protein kinase involved in cell division and proliferation, its overexpression being frequently associated with carcinogenesis; in addition, high PIk1 levels are correlated to the inactivation of p53 tumor suppressor in cancer cells [224]. UA was able to induce autophagy and apoptosis in T47D, MCF-7, and MDA-MB-231 human breast cancer cell lines by suppressing the PI3K/AKT pathway and leading to the down-regulation of B-cell lymphoma 2 and the up-regulation of glycogen synthase kinase activity, which further stimulated caspase-3 and inhibited cyclin-D1. Moreover, UA was able to stop breast cancer progression by inhibiting the inflammatory processes modulated by NF-kB; the phytocompound blocked the phosphorylation of NF-kB by inhibiting the expression of IKKα following the suppression of pro-inflammatory cytokines (TNF-α, IL-6, IFN-γ), thus ameliorating the inflammatory response in breast cancer cells [225]. UA may act as cytotoxic agent via Nrf2 pathway inhibition; Zhang et al. have reported that UA suppressed the Nrf2- mediated cell proliferation regulated by EGFR and Keap1 in MDA-MB-231 breast cancer cells in a dose-dependent manner. Nrf2 is a transcription factor with an important role in oxidative and electrophilic stress; under stress conditions Nrf-2 is dissociated from keal-like ECH-associated protein 1 (Keap 1) and translocated to the nucleus in order to enhance the antioxidant response [226]. A huge challenge in the management of breast cancer treatment with placlitaxel is the development of drug resistance due to several deactivation pathways. Xiang et al. reported that UA is able to reverse paclitaxel resistance by targeting the miRNA-149-5p/MyD88 pathway [227]. The authors had previously analyzed the implication of miR-149-5p and MyD88 levels in paclitaxel chemoresistance [228] and revealed that miR-149-5p plays a critical role in preventing chemoresistance; it directly targeted MyD88 and down-regulated its expression, proving to be a key regulator in macrophage TLR/MyD88 signaling pathway. UA has the ability to induce an in vitro apoptotic effect by up-regulating the expression of miR-149-5p and down-regulating the expression of MyD88, hence reversing chemoresistance and acting as a potential future paclitaxel co-treatment agent against breast cancer [227]. Another study exploring UA’s ability to overcome drug resistance was conducted by Lin et al., who assessed the cytotoxic effect of UA on gemcitabine-resistant human pancreatic cancer cells. The molecular mechanism behind the UA’s preventive activity against drug resistance consists in modulating the expression of the receptor for advanced glycation end products (RAGE), which was reported to be overexpressed in gemcitabine resistance. Furthermore, UA induced autophagy and apoptosis in a dose-dependent manner in human pancreatic cancer MIA Paca-2 and MIA Paca-2GEMR cell lines through cell cycle arrest and endoplasmatic reticulum (ER) stress. In addition, the ER stress induced by UA promotes the up-regulation of proapoptotic Bax protein expression and the down-regulation of anti-apoptotic Bcl-2 protein, hence inducing apoptosis in both cell lines [229].

Kim et al. reported that UA suppressed the invasion and migration rates of SNU484 and SNU638 gastric cells in a dose-dependent manner by regulating the Hippo pathway through promoting its upstream target genes (MST1, MST2, LATS1), and Ras association domain family (Rassf1). The Hippo pathway is responsible for cell growth and survival; after being activated, it displaces phosphorylated Yap in the cytoplasm, leading to cell degradation, hence stopping cell proliferation; the experimental data also showed that UA was able to inhibit tumor growth in xenograft animals [230].

Skin cancer can also be targeted by UA, which activates the peroxisome proliferator activated receptor α (PPARα) and AMPK in mouse squamous cell carcinoma Ca3/7 cells and mouse skin papilloma MT1/2 cells [230]. PPARα has the ability to bind to DNA with Retinoid X Receptor (RXR) as a heterodimer that recognizes specific genes; this process is promoted by agonist ligands, which regulate the gene expression of other transmission factors through protein interactions. The activation of PPARα by UA, however not through direct binding, led to the apoptosis of skin cancer cells that were previously treated with PPARα and AMPK inhibitors [231]. Within the field of skin cancer, the genomic and epigenomic mechanisms responsible for UA activity on UVB-induced non-melanoma skin cancers were investigated through Cp-methyl sequencing and RNA sequencing; it was reported that UA regulates the IL-6, NF-kB, and Nrf2 inflammatory pathways as well as cell cycle regulatory pathways [232].

When tested against cervical cancer, UA exhibited a strong apoptotic effect both in vitro on CaSki, HeLa, C4-1, and SiHA human cervical cell lines and in vivo on xenograft tumor models. In terms of molecular mechanisms, UA up-regulated p53 and pro-apoptotic Bax protein and down-regulated the expression of anti-apoptotic Bcl-2 protein and protein 1 (cIAP1) apoptotic inhibitor, thus inducing apoptosis and cell death (Figure 14) [233]. Cellular inhibitor of apoptosis protein-1 (cIAP1) belongs to the anti-apoptotic protein family IAP, whose overexpression promotes cancer cell survival under stress conditions [234].

The L858R/T790M mutation of the epidermal growth factor receptor (EGFR) represents a major cause of EGFR treatment in non-small-cell lung (NSCLC) carcinoma. Yang et al. studied the effect of UA on overcoming the challenging treatment of NSCL by mediating the CT45A2 gene transcription. CT45A2 is a proto-oncogene that belongs to the CT45 family and has an important role in tumorigenesis. The results showed that CT45A2 was up-regulated in H1975 NSCLC cells and expressed EGFR with the T790M mutation. Furthermore, UA inhibited the transcription factor TCF4, that plays an important role in CT45A2 gene transcription, by down-regulating the β-catenin/TCF4 transcriptional activity through blocking the nuclear translocation of β-catenin, hence inhibiting the proliferation and motility of NSCLC cells [235].

UA exhibited a ROS-dependent autophagy in esophageal squamous cell carcinoma (ESCC) TE-8 and TE-12 cell lines previously treated with ROS inhibitors; the authors reported that UA reduced cell migration and invasion and induced apoptosis and autophagy in a dose-dependent manner. In terms of molecular mechanisms, UA induced ESCC apoptosis by enhancing the levels of cleaved PARP and caspase-9 proteins, resulting in sub-G1 cellular cycle phase arrest. Furthermore, UA modulated the Akt/mTOR pathway, which resulted in increased ROS production by up-regulating the LC3-II levels and down-regulating the p62 protein levels, finally resulting in ESCC autophagy [236].

The correlation between abnormal levels of cholesterol and different types of cancer has been widely explored; more specifically, the expression of sterol regulatory element-binding protein 2 (SREBP2) has been correlated with the evolution of hepatocellular carcinoma. Kim et al. highlighted the apoptotic effect of UA against HCC through the modulation of cholesterol supplementation; at low intracellular cholesterol levels, SREBP2 initiates a transcriptional programming through the activation of target genes, inducing de novo cholesterol synthesis, which promotes growth signaling factors such as PI3K/AKT and EGRF/MAPK. When intracellular cholesterol levels are elevated, SREBP2 is not processed to maturation and cholesterol synthesis is not stimulated. UA activates SREBP2, thus inducing cell cycle arrest and apoptosis in HCC cells by inhibiting phosphorylation of the oncogenic AKT and MAPK pathways [237].

### 4.2. Antidiabetic Activity

Reactive oxygen species play an important role in the development of various congenital disorders such as those caused by gestational diabetes; high levels of ROS in mothers with prenatal diabetes can lead to DNA or RNA harm in the embryos. Dai et al. analyzed the antioxidant effect of UA in pregnant rats with pre-induced streptozotocin gestational diabetes; the underlying molecular mechanism resides in the modulation of AGEs-RAGE signaling pathway [238], which has been thoroughly studied in several pathologies, particularly in diabetes where it increases oxidative stress by activating pro-inflammatory mediators such as NOX -1, TGF-β, NFkB, and ERK1/2 and down-regulating the SOD-1 expression [239]. UA dose-dependently inhibits the development abnormalities and biochemical parameters of diabetic rats fetuses compared to the control group [238]. The antidiabetic activity of UA had been previously documented by in vitro and in vivo studies that showed a strong inhibitory effect on the two main glucosidases, α-amylase and α-glucosidase, thus resulting in an obvious hypoglycemia. Molecular docking revealed that UA inhibits the two glucosidases by bonding to their inactive sites through hydrogen bonds and subsequently down-regulating their activity [240].

Diabetic cardiomyopathy is one of the most common complications in poorly conducted diabetes treatment with patients exhibiting left ventricular dysfunction and cardiomyocyte hypertrophy; UA revealed the ability to alleviate oxidative stress and inflammatory processes in diabetic cardiomyopathic rats. It inhibits the production of pro-inflammatory cytokines TNF-α, MCP-1 and TGF-β1 and up-regulates MMP-2 protein levels, leading to attenuated myocardial injuries such as cardiac fibrosis [241].

### 4.3. Antiinfectious Activity

Zoonotic infections have become significantly widespread around the globe; *Toxoplasma gondii* infections occur in many countries and have become a risk since they cause serious consequences in all age groups, particularly in pregnant women. Choi et al. reported that UA inhibited the expression of IL-1β, IL-6, TNF-α, and TGF-β1 pro-inflammatory cytokines, and stimulated the anti-inflammatory cytokines IL-10, Il-12, and granulocyte macrophage colony stimulating factor (GM-CSF) as well as the production of NO and ROS in parasite-infected immune cells. In addition, the phytocompound inhibited *T. gondii* proliferation by down-regulating the expression of ROP 18, MIC 8, and IMC sub 3 subcellular organelles in *T. gondii*-infected cells; therefore, one may state that UA acts as an antiproliferative agent against *T. gondii* while simultaneously modulating the immune response in parasite-infected immune cells [242]. UA could be used as a novel therapy in visceral leishmaniasis due to the inhibition of IL-4 proinflammatory cytokine, which in turn was not able to activate the STAT-6 transcription factor; subsequently, the NO production–which is essential in parasite elimination–remained unaltered [243]. IL-4 is a cytokine with an important regulatory role in immunity; IL-4 signaling is mediated by the IL-4 receptor alpha chain (IL-4Rα) that dimerizes after binding to a ligand, leading to type 1 signaling complex activation, which is phosphorylated by Janus family kinases, finally leading to the activation of STAT-6 transcription factor [244], able to inhibit NO production and to stop parasite elimination. 

Quian et al. examined UA’s antimicrobial activity against carbapenem-resistant *K. pneumoniae* (CRKP) that represents a serious public health threat; the results showed that UA was able to alter bacterial membrane integrity and inhibit biofilm formation, hence providing a potential future treatment against CRKP [245]. 

The disturbance of membrane permeability and integrity can be induced by UA in fungi as well; such an example is *Alternaria alternata*, a latent fungus that contaminates several types of fruits and whose metabolites can be detrimental to humans in which UA up-regulates ROS production and accumulation, leading to pathogen lysis [246].

### 4.4. Anti-Atherosclerotic Activity

UA was found active against phenylephrine-induced right ventricle hypertrophy in neonatal rats model; the report showed that UA regulated the PPARα-dependent fatty acid metabolism through the up-regulation of PPARα and CPT1b genes expressions which are down-regulated in hypertrophy, resulting in the alleviation of tissue fibrosis, cell apoptosis and metabolic abnormalities [247].

Qui et al. have investigated the anti-atherosclerotic effect of UA both in vitro and in vivo; UA activity was explained by the up-regulation of lecitin-like oxidized low-density lipoprotein receptor 1 (LOX-1) expression and the down-regulation of the TLR4/MyD88 signaling pathway. Moreover, UA inhibited the NF-kB pathway, leading to the suppression of ROS production [248]. LOX-1 is a transmembrane protein highly expressed in macrophages, vascular smooth cells, and endothelial cells; after being activated by OxLDL, it up-regulates the NF-kB signaling pathway, leading to increased ROS formation and the subsequent induction of adhesion molecules and endothelial apoptosis. LOX-1 overexpression is frequently associated with pro-atherogenic settings, making it a suitable target for treating atherosclerosis [249]. TRL4 is a well-studied member of the TRL family, which together with myeloid differentiation primary response 88 (MyD88) are activated by lipopolysaccharides through direct binding; in turn, TRL4/MyD88 up-regulates the expression of NF-kB, leading to the activation of pro-inflammatory genes and releasing pro-inflammatory cytokines [250]. The inhibition of the TRL4/MyD88 pathway induced by UA through the up-regulation of Beclin-1 protein expression leads to enhanced macrophage autophagy and reduced inflammatory processes both in vitro and in vivo [251].

UA showed an efficient activity against in vitro leptin-induced atherosclerosis in rat vascular smooth muscle cells; in terms of molecular mechanism, UA exhibited an inhibitory effect on ERK1/2 activation, NF-kB expression, and leptin-induced MMP2 activity, hence decreasing ROS production. The down-regulation of MMP2 secretion by UA induced the inhibition of MAPK/ERK signaling pathway, hence inhibiting cell proliferation and atherosclerosis [252].

### 4.5. Neuroprotective Activity

In a similar manner than its isomer OA, UA exerts strong neuroprotective effects against traumatic brain injuries in mice by activating the Nrf2 pathway. Under physiological conditions, Nrf2 is bound to the Kelch-like ECH associated protein (Keap1) where it is subject to degradation; however, under pathological conditions, Nrf2 is released into the nucleus and binds to antioxidant response element (ARE), leading to the activation of downstream protective factors such as heme oxygenase 1 (HO1) and NADPH quinine oxido-reductase 1 (NQO1), which regulate redox balance. Experimental results showed that UA was able to activate the Nrf2 pathway, therefore up-regulating anti-oxidant enzymes, reducing oxidative stress, and producing neuroprotective effects [253]. 

UA was able to mimic human natural killer-1 (HNK-1) properties in C57BL/6J mice models of spinal cord injury where the phytocompound inhibited the pro-inflammatory markers; HNK-1 is a glycan epitope involved in several neural functions, including the regeneration of the nervous system. The results showed that UA down-regulated MAPK and PI3K/AKT/mTOR pathways, which are activated in the acute phase of inflammation, leading to the suppression of proinflammatory markers such as IL6 and TNF-α; subsequently, it stimulated the structural remodeling for injury regeneration [254].

Wang et al. indicated that the anti-inflammatory effect of UA in alleviating cerebral ischemia and reperfusion injury is accomplished by inhibiting the HMGB1/TRL4/NF-kB pathway and causing the inhibition of TNF-α, IL-1β, and IL-6 pro-inflammatory cytokines; subsequently, UA is able to attenuate ischemia and neuronal apoptosis [255].

### 4.6. Antiinflammatory Activity

Mou et al. reported that UA was able to ameliorate simulated autoimmune thyroiditis in human follicular epithelial cells (Nthy-ori 3-1) by inhibiting the MALAT1/miR-206/PTGS1 and NF-kB signaling pathways. MALAT1 possesses a key role in cellular proliferation and apoptosis; miR-206 is an important member of the MIR gene family, acting as a crucial regulator in muscular differentiation and neuromuscular junction repair while PTGS1 is an enzyme that regulates prostaglandin synthesis in various organs; collectively, the MALAT1/miR-206/PTGS1 pathway is deeply involved in inflammatory processes. UA alleviated IL-1β induced Nthy-ori 3-1 cell damage by down-regulating MALAT1/miR-206/PTGS1 and NF-kB expression, hence inhibiting the pro-inflammatory cytokines [256].

The anti-inflammatory effect of UA was investigated in cigarette smoke-induced emphysema in rats; the authors have reported that UA was able to alleviate the emphysema severity by modulating the PERK pathway and decreasing 8-OHdG and MDA oxidative stress factors. The phytocompound inhibited the activation of PERK, leading to the down-regulation of Bax pro-apoptotic protein and up-regulation of Bcl-2 anti-apoptotic protein; furthermore, UA was able to inhibit the inflammatory caspase 3 and caspase 9, and stimulate the expression of the Nrf2/ARE pathway, thus alleviating experimental emphysema [257]. The authors have further investigated UA’s ameliorating effects on muscle consumption in cigarette smoke-induced emphysema; the results showed that UA up-regulated IGF-1 and down-regulated the TGF-β1/Smad 2,3 pathway, leading to the suppression of EMT and EndMT, thus alleviating airway–vessel remodeling and muscle atrophy [258]. Additional anti-inflammatory mechanisms were reported by Ma et al., who conducted in vitro studies on BEAS-2B cells; the phytocompound inhibited TNF-α and IL-1β expression, leading to the subsequent inhibition of caspase 3 activation, which in turn down-regulated the expression of NF-kB, IL-6 pro-inflammatory cytokine and NO release. Furthermore, UA was able to inhibit STAT-3 expression by down-regulating its target genes, such as cyclin D, Bcl-2, Bcl-xL, and VEGF, hence demonstrating a potential future therapeutic use against inflammatory diseases [259]. Similar molecular mechanisms were reported in UV-B radiation-induced photoaging in human skin dermal fibroblasts, thus showing UA’s significant potential to mitigate UVB-induced extracellular damage and to act as a skin protective agent [260]. 

UA exhibited a suppressive effect against muscle wasting in induced chronic kidney disease both in vitro and in vivo; the phytocompound down-regulated the expression of myostatin and TNF-α, IL-1β, and IL-6 pro-inflammatory cytokines, led to the inhibition of ubiquitin E3 ligase, and decreased proteolysis [261]. Complementarily, UA counteracts hypobaric hypoxia-induced skeletal muscle protein loss through the up-regulation of the Akt signaling pathway, thus stimulating protein synthesis [262].

Kim et al. have explored the alleviating effect of UA in rheumatoid arthritis in RASF (Rheumatoid arthritis synovial fibroblast); UA facilitated RASF apoptosis by down-regulating the expression of myeloid cell leukemia-1 (Mcl-1) through the enhancement of Noxa selective binding to Mcl-1. Mcl-1 belongs to the anti-apoptotic Bcl-2 family, playing a key role in intrinsic and extrinsic cell death signals and enhancing apoptosis resistance [263]. 

### 4.7. Other Biological Activities

The ability of UA to ameliorate induced-nonalcoholic fatty liver was explored through modulating the LXRα receptor on a T090 induced mouse model; the results showed that the phytocompound significantly decreased the LXR response element by competitive binding; furthermore, UA up-regulated AMPK phosphorylation in hepatic cells, but down-regulated the SREBP-1 promoter region, leading to reduced hepatic and cellular contents [264]. The phytocompound also acted as an alleviating agent in ochratoxin A-induced renal cytotoxicity; the molecular mechanism resides in UA’s inhibitory activity on ROS, by inhibiting the expression of Lon protease 1 (Lonp1), Aco2, and Hsp75, hence suppressing apoptosis and cell death. Lonp1 is a key protein that regulates oxidative stress and cell survival by contributing to mitochondrial processes such as DNA maintenance, mitochondrial protein control, and unfolded protein response; its down-regulation is correlated to reduced expressions of OXPHOS complexes [265].

## 5. Concluding Remarks and Future Perspectives

Triterpenic acids have been found to exert a plethora of pharmacological activities such as anticancer, anti-inflammatory, antidiabetic, antioxidant, cardioprotective, hepatoprotective, neuroprotective, antimicrobial, etc. Their potential efficacy needed to be fully explored due to their pharmacological selectivity that prevents the occurrence of hardly tolerable side effects compared to the synthetic drugs used currently in therapy. In the last decades, many underlying molecular mechanisms were identified and described; however, in order to fully understand the best formulation options for triterpenic acids and to thus achieve their highest therapeutic potential, further studies must be conducted since–despite the huge progresses in the area–there are still many aspects to be revealed at the molecular level. Such investigations are crucial for future clinical trials and in order to reveal important information regarding the compound’s safety and posology, preventing late-stage failure in drug development.

Our work aimed to summarize the molecular mechanisms involved in the pharmacological activities of three intensively researched triterpenic acids: AA, OA, and UA. The literature survey on these phytocompounds revealed a variety of signaling pathways that trigger specific pharmacological effects both in vitro and in vivo. Some studies, however, reported contradictory results, which might lead researchers to the conclusion that the pharmacological profile of a phytocompound is the result of triggering a combination of molecular pathways in a dose-dependent manner. This knowledge might contribute to future research that aim to develop triterpenic acids-based drugs that may improve the current therapeutic arsenal.
